# Crystal twinning of bicontinuous cubic structures

**DOI:** 10.1107/S2052252519017287

**Published:** 2020-02-01

**Authors:** Lu Han, Nobuhisa Fujita, Hao Chen, Chenyu Jin, Osamu Terasaki, Shunai Che

**Affiliations:** a School of Chemical Science and Engineering, Tongji University, Shanghai 200092, People’s Republic of China; b Institute of Multidisciplinary Research for Advanced Materials, Tohoku University, Sendai 980-8577, Japan; c JST, PRESTO, Saitama 332-0012, Japan; d Institut für Numerische und Angewandte Mathematik, Georg-August-Universität Göttingen, Lotzestr. 16-18, Göttingen 37083, Germany; e Max Planck Institute for Dynamics and Self-Organisation, Am Faßberg 17, Göttingen 37077, Germany; f Centre for High-resolution Electron Microscopy, School of Physical Science and Technology, ShanghaiTech University, Shanghai 201210, People’s Republic of China; g Department of Materials and Environmental Chemistry, Stockholm University, Stockholm S-10691, Sweden; h School of Chemistry and Chemical Engineering, Shanghai Jiao Tong University, Shanghai 200240, People’s Republic of China

**Keywords:** twinning defects, electron crystallography, bicontinuous cubic structures, constant mean curvature surfaces, inorganic porous solids, crystal distortions

## Abstract

Bicontinuous cubic structures in soft matter consist of two intertwining labyrinths separated by a partitioning layer. Combining experiments, numerical modelling and techniques in differential geometry, we investigate twinning defects in bicontinuous cubic structures. We first demonstrate that a twin boundary is most likely to occur at a plane that cuts the partitioning layer almost perpendicularly, so that the perturbation caused by twinning remains minimal. This principle can be used as a criterion to identify potential twin boundaries, as demonstrated through detailed investigations of mesoporous silica crystals characterized by diamond and gyroid surfaces. We then discuss that a twin boundary can result from a stacking fault in the arrangement of inter-lamellar attachments at an early stage of structure formation. It is further shown that enhanced curvature fluctuations near the twin boundary would cost energy because of geometrical frustration, which would be eased by a crystal distortion that is experimentally observed.

## Abstract 2

State-of-the-art electron crystallography when combined with geometric techniques to model curved surfaces enables an in-depth study of twinning defects in bicontinuous cubic structures.

## 1. Introduction   

Bicontinuous cubic structures (BCSs) are crystal structures with cubic symmetry consisting of two continuous intertwining subvolumes, or labyrinths, separated by a non-self-intersecting partitioning layer. They are found in cell endomembrane systems (Landh, 1995 ▸), butterfly wing scales and beetle exo­skeletons (Galusha *et al.*, 2008 ▸; Michielsen & Stavenga, 2008 ▸), lyotropic liquid crystals (LLCs) and related systems [mesoporous silica crystals (MSCs), block copolymer self-assemblies, *etc*.] (Luzzati & Spegt, 1967 ▸; Hyde *et al.*, 1984 ▸; Alward *et al.*, 1986 ▸; Kresge *et al.*, 1992 ▸; Bates & Fredrickson, 1999 ▸; Wan & Zhao, 2007 ▸). Such structures are known to possess unique properties, such as structural colours (Galusha *et al.*, 2008 ▸; Michielsen & Stavenga, 2008 ▸), unusual mechanical and electronic features (Bruinsma, 1992 ▸; Fujita & Terasaki, 2005 ▸), and biological functions (Ellens *et al.*, 1989 ▸; Caffrey, 2000 ▸; Shah *et al.*, 2001 ▸).

Different sides of the partitioning layer can be filled with mutually immiscible sub-chains in block copolymer systems, whereas the two labyrinths of a BCS in LLCs are homophilic. In the latter, the partitioning layer comprises thinner sublayers of counter components and is bounded by the hydro­philic/hydro­phobic interfaces of amphiphilic molecules. The partitioning layer tends to follow triply periodic minimal surfaces (TPMSs). The continuous nature distinguishes BCSs from conventional crystals, which are usually described as a discrete arrangement of constituents (*e.g.* atoms). The formation of BCSs in LLCs (Schwarz & Gompper, 2000*a* ▸,*b* ▸) is currently explained in terms of bending and stretching energies associated with the hydro­philic/hydro­phobic interfaces, following the work by Helfrich (Helfrich, 1973 ▸), leading to two interpretations of the partitioning layer: either bounded by a pair of parallel surfaces with fixed distance off the TPMS, or by a pair of constant mean curvature (CMC) companions of the TPMS. The mean curvatures for the two CMC companions are equal in magnitude but opposite in sign. While the parallel surface model is more straightforward (Shearman *et al.*, 2007 ▸), competition and compromise between the two models have only recently been scrutinized (Chen & Jin, 2017 ▸).

Crystal defects, *e.g.* twinning (Cahn, 1954 ▸), often carry a wealth of information on crystal growth and structural transformation. Contact reflection twins, a common form of twinning, involve two individual crystalline domains with an identical structure related by a reflection in their common boundary plane (termed twin boundary). Hereafter, the term ‘twinning’ will only refer to contact reflection twins unless otherwise stated. Despite its importance, twinning in BCSs has rarely been considered until recently.

MSCs are fabricated by the cooperative self-assembly of an LLC system with silicates as inorganic precursors (Kresge *et al.*, 1992 ▸; Wan & Zhao, 2007 ▸). In MSCs, BCSs are often formed as an intermediate structure between cylindrical and lamellar structures (Kaneda *et al.*, 2002 ▸; Gao *et al.*, 2006 ▸; Han *et al.*, 2009 ▸), where the silica wall traces the partitioning layer in the LLC phase before calcination. Recently, Han *et al.* (2011 ▸ *b*) reported a BCS twinning in spherical MSC particles with polyhedral cavities. More specifically, each facet of the cavity is a {111} surface of a single domain of a BCS with the geometry of a diamond (D) surface (termed D-BCS), while a twin boundary is observed at each polyhedral edge that bounds adjacent facets. More recently, a similar twinning has also been observed in block copolymer systems (Lin *et al.*, 2017 ▸). So far, however, no common law is known for describing the structure, formation and stability of twin boundaries in BCSs, as the conventional description of twins for discrete atomic crystals does not apply. The main difference here lies in the fact that BCSs are continuous structures that can be cut through by an arbitrary plane, in sharp contrast to conventional crystals.

Herein, a principle of BCS twinning is described in detail for the first time: a twin boundary should intersect the partitioning layer of an un-twinned BCS almost perpendicularly, so that the BCS is minimally perturbed by the twinning. This principle is verified through analysing the observed twinning of D-BCS, termed D twin, in an MSC using electron microscopic techniques (Han *et al.*, 2011 ▸ *b*) and may serve as a practical criterion for preliminarily locating potential twin boundaries in general. It has been successfully applied to single out an observed twin boundary in another kind of BCS with the geometry of a gyroid (G) surface, termed G-BCS. Moreover, we point out that a D-twin boundary can be thought of as a stacking fault in a layered stacking of catenoidal channels, providing further insights into the formation of twin boundaries. Finally, analysis of Gaussian curvatures reveals how twin boundaries, as microscopic topological defects, may be the cause of crystal distortions manifested in the macroscopic morphology of twinned BCSs.

## 2. Crystallographic reconstruction of twinned MSCs   

A twin boundary in conventional crystals can be described as the corresponding atomic plane specified by Miller indices. In view of the continuous nature of BCSs, we define an additional offset parameter *x* as a fraction of *d_hkl_*, the *d*-spacing of the {*hkl*} planes, to indicate the relative position of a twin boundary with respect to the reference planes. Hence, the twin boundary is specified as {*hkl*} + *x*. The reference plane with *x* = 0 is taken to include flat points on the associated TPMS. For the D surface, for which the space group of the non-oriented [oriented] TPMS is 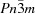 [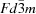], the flat points correspond to the Wyckoff positions 4*b* [32*e*] with the site symmetry 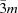 [3*m*]. On the other hand, the flat points for the G surface, for which the space group is 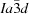 [*I*4_1_32], take the Wyckoff positions 16*a* [16*e*] with the site symmetry 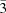 [3]. Note that the surface normal vectors at the flat points are parallel to 〈111〉 in either case. Generally speaking, the reference {*hkl*} plane may not be determined uniquely by the flat points. In the case of the G surface, in particular, the flat points can be classified according to whether their normal vectors are parallel to the {211} twin boundary or not. This in fact gives us two distinct choices of the reference {211} plane to describe a twin boundary of G-BCS. In this work, the reference plane is chosen such that it includes only those flat points whose surface normal vectors are not parallel to the plane and hence not normal to the plane normal 〈211〉.

**Figure 1:**
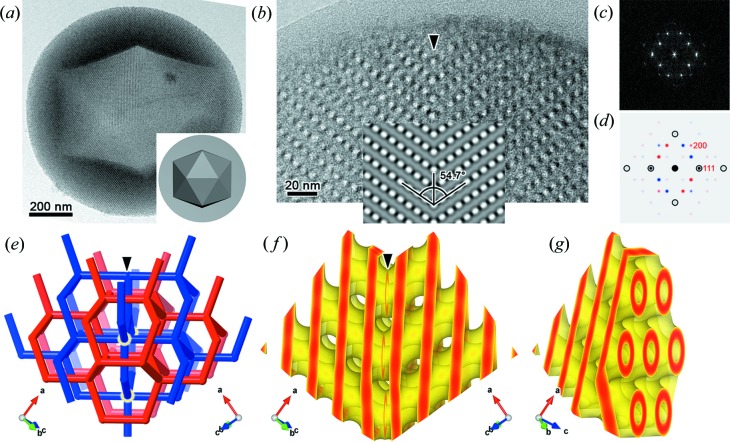
HRTEM image and structural models of a D-twin boundary. (*a*) A low-magnification TEM image of a spherical particle with an icosahedral cavity in the centre, where a schematic illustration is given in the inset. (*b*) An HRTEM image of a sliced sample taken from the common [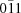] axis. The inset shows a hypothetical image obtained by mirroring a simulated HRTEM with an offset value of 0.5, showing that the twin boundary is {111} + 0.5. The white contrasts correspond to regions with low electrostatic potential. (*c*) The Fourier transform of the HRTEM image shown in (*b*). (*d*) A superposition of two simulated ED patterns (red and blue), where the 111 reflections of both domains overlap with each other (black). (*e*) The topology of a D-twin boundary shown as skeletal graphs, in which the 5-connected vertices are highlighted with yellow circles. (*f*) A 3D model of the D-twin boundary obtained by joining two 3D reconstructed copies, shown in (*g*), at {111} + 0.5.

Thanks to the stability of the inorganic skeletons under electron beam, MSCs are amenable to detailed structural analyses. A transmission electron microscopy (TEM) image of a typical MSC particle with multiply twinned D-BCS (Han *et al.*, 2011*b* ▸) is shown in Fig. 1 ▸(*a*). Fine details of the twin boundaries are revealed through high-resolution TEM (HRTEM) observations on sliced samples embedded in ep­oxy resin. Fig. 1 ▸(*b*) shows two structural sub-domains, each of which show the typical contrast from 〈110〉 directions of D-BCS. Here, alternating bright and dark contrast signifies the projection of electron-scattering intensities in the silica wall. The sub-domains are the twin individuals contacting each other at a {111} plane (running vertically) whose contact angle with one of the {100} planes is 54.7°. Accordingly, the Fourier transform [Fig. 1 ▸(*c*)] of the HRTEM image shows two sets of diffraction spots, in which the 111 reflections overlap with each other. To decide the offset of the twin boundary, an HRTEM image and an electron diffraction (ED) pattern of a twin individual were simulated using the software *MesoPoreImage* (Ohsuna *et al.*, 2011 ▸) using a 3-term nodal approximation (Gandy *et al.*, 2001 ▸). The simulated HRTEM image was then combined with its mirror image at different offsets (see Fig. S2 in the Supporting information); the experimental contrast is best reproduced with a twin boundary at {111} + 0.5 [inset of Fig. 1 ▸(*b*)]. The geometrical relationship in Fig. 1 ▸(*c*) is clarified in Fig. 1 ▸(*d*) where simulated ED patterns for the twin individuals are superposed. (Note, the Fourier transform of a TEM image is generally affected by the contrast transfer function while an ED pattern is not, so their peak intensities are not directly related.) The two labyrinths can be sketched using skeletal graphs with 4-connected nodes at the 2*a* positions for the space group 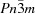 with the site symmetry 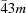. Around the twin boundary, the red skeleton maintains the 4-connectivity, while the blue skeleton involves 5-connected nodes at the boundary because of a modified topology [Fig. 1 ▸(*e*)].

Han *et al.* (2011*a* ▸,*b* ▸) reconstructed an average silica wall structure within an individual domain of D-BCS using an electron crystallographic technique (Sakamoto *et al.*, 2000 ▸; Miyasaka & Terasaki, 2010 ▸; Willhammar *et al.*, 2012 ▸). Here, the structure factors extracted from a series of HRTEM images taken along high-symmetric zone axes were used to obtain the electrostatic potential map. Then, the side surfaces of the silica wall were determined as the equipotential surfaces that minimize an energy density associated with the curvature. To be specific, the Helfrich free-energy density, *f* = κ_1_(〈*H* ^2^〉 − 〈*H*〉^2^) + κ_2_〈*K*〉, was employed, where *H* and *K* are the mean and Gaussian curvatures, respectively, and 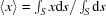 is the area-weighted average of *x* on the surface *S*. Although it is difficult to determine the two coefficients, κ_1_ and κ_2_, *a priori*, the authors heuristically assumed κ_2_ = 0, thereby only the mean curvature fluctuations contribute to the energy, as if the side surfaces favoured CMC surfaces. (If 〈*H*〉 is identified with a predefined constant *H*_0_, 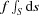 reduces to the modified Willmore functional to be discussed later.) The threshold of the equipotential was thus determined at 75% within the min–max range of the electrostatic potential map (see Table S1 and Fig. S3 in the Supporting information). Remarkably, this crude approximation could fairly reproduce the high-resolution electron micrographs [Fig. 1 ▸(*b*)]. The reconstructed silica wall domain [Fig. 1 ▸(*g*)] is now mirrored at {111} + 0.5 to mimic the D-twin boundary [Fig. 1 ▸(*f*)].

## 3. In favour of smoothness   

The D-twin model above, obtained by simply putting two reconstructed D-BCS together [Fig. 1 ▸(*f*)], seems fairly smooth although not quite, suggesting that the silica wall in the un-twinned BCS intersects with the twin boundary almost perpendicularly. Only a small perturbation seems necessary to restore smoothness. In other words, for the preference of minimal perturbation, a potential twin boundary should, in general, intersect the BCS partitioning layer almost perpendicularly.

**Figure 2:**
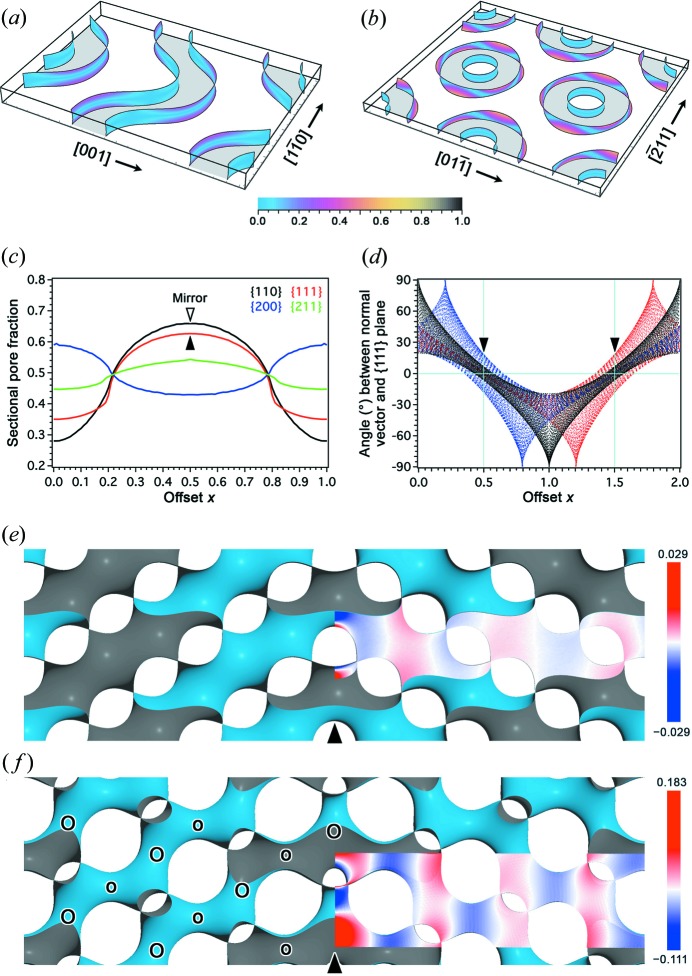
Structural assessment of a D-twin boundary. (*a*), (*b*) Rendering of |**n**_s_·**n**_b_| on the side surfaces of the reconstructed silica wall at {110} + 0.5 and {111} + 0.5, respectively, shown within slab regions of thickness 0.2 in units of the relevant *d* spacings. (*c*) Sectional pore fractions calculated with the reconstructed silica wall, in which the {110} + 0.5 plane shows the highest contrast followed by the {111} + 0.5 plane. (*d*) Scatter plot of elevation angles of the surface normal vectors with respect to {111} planes plotted against the offset values, for the D surface (black) and its CMC companions (red and blue). (*e*), (*f*) Twin-boundary sections of polysynthetic D-twins constructed as minimal and CMC surfaces, respectively, viewed along a common 〈110〉 direction. Colour maps are overlaid to show the minimal distances in units of the *d* spacing from the un-twinned D surface, where deviations in opposite senses are shown in blue and red while coincidence is shown in white. In (*f*), small and large necks, as indicated by the ‘o’ and ‘O’ symbols, alternate along the boundary normal.

The perpendicularity of a surface with a plane can be quantified by the dot products |**n**_s_·**n**_b_| over the intersection, where **n**_s_ denotes the unit normal vectors of the surface and **n**_b_ denotes the unit normal vector of the plane. By checking the distribution of |**n**_s_·**n**_b_| slice by slice throughout the reconstructed D-BCS along different crystal axes, we find that the {110} + 0.5 plane is perfectly perpendicular to the equipotential surfaces [Figs. 2 ▸(*a*) and S4]. This is no surprise since it is actually a mirror plane in D-BCS. The second-best candidate is the {111} + 0.5 plane [Figs. 2 ▸(*b*) and S5], which corresponds to the observed twin boundaries.

The D surface and its CMC companions may also be used to approximate the median and side surfaces of the partitioning layer of D-BCS. These surfaces can be numerically constructed using Brakke’s *Surface Evolver* (Brakke, 1992 ▸), an efficient gradient descender (see Appendix *A* [App appa]), and lend themselves to geometrical analyses. The elevation angles of surface normal vectors with respect to {111} at uniformly sampled points on the surfaces are plotted in Fig. 2 ▸(*d*), which again confirms that {111} + 0.5 is the most preferable in terms of perpendicularity. It is instructive to note that the plots in Fig. 2 ▸(*d*) reflect symmetry elements of the surfaces. Because of the inversion symmetry, the black dots are distributed symmetrically with respect to the vertical lines at integer offsets (*e.g.* *x* = 0), which correspond to the planes containing flat points (*i.e.* inversion centres), whereas the same vertical mirrors exchange the blue and red dots corresponding to the two CMC companions. Also, the distribution of dots of each colour is symmetric with respect to any half-integer offset point (*e.g.* *x* = 0.5) on the horizontal axis (*i.e.* the zero angle line) since twofold rotational axes lie in the relevant {111} plane.

Generally speaking, twin boundaries tend to favour low indices. In the case of conventional crystals, twin boundaries usually correspond to dense atomic planes costing low boundary energies. This naturally makes the density variation high along the normal axis to the twin boundary. In the case of BCS twinning, the perpendicularity of the partitioning layer leads to high sectional pore fractions instead [Figs. 2 ▸(*c*) and S6]. This has two implications. On the one hand, a high pore fraction indicates flexibility of the structure subject to perturbation; on the other hand, it also results in a high density variation along the boundary normal. (The projected density onto an axis through the origin is directly connected with X-ray structure factors lying on the same axis, whereas corresponding electron-structure factors are somewhat more loosely correlated.) For our D-BCS, the strongest reflection is {110}, associated with the mirror plane; the second strongest is {111}, associated with the observed D-twin boundary (Fig. S1 and Table S1). In order to identify potential twin boundaries, it is therefore important to compare the perpendicularities of different planes that show large density variations along their normal axes.

## 4. Numerical modelling of minimal and CMC twins   

We now investigate structural perturbations caused by the twinning defects by constructing twinned minimal and CMC surfaces using *Surface Evolver* (Brakke, 1992 ▸). A single twin boundary is approximated by parallel twin boundaries at a distance comparable to the experiment. Free boundary conditions are imposed on the boundary planes so that the surface can be extended through reflection. The surface integral of (*H*−*H*_0_)^2^, where *H* is the mean curvature and *H*_0_ is a prescribed mean curvature, can be used as the cost function such that a surface with a mean curvature *H*_0_ is obtained if it is numerically reduced down to 0 (Große-Brauckmann, 1997 ▸). The latter integral is a modification of the so-called Willmore functional (Hsu *et al.*, 1992 ▸), for which the integrand is *H* ^2^. Figs. 2 ▸(*e*) and 2 ▸(*f*) show constructed minimal (*H*_0_ = 0) and CMC (*H*_0_ = 1.1/*a*_D_) surfaces, respectively, for modelling a D-twin boundary, where *a*_D_ is the cubic lattice constant of the D surface under the space group 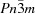.

The perturbation caused by twinning is shown in the overlays of Figs. 2 ▸(*e*) and 2 ▸(*f*), where blue and red indicate the normal deviations of the twinned surface from its un-twinned original in opposite senses as drawn using the *CloudCompare* package (Girardeau-Montaut, 2015 ▸). Note that the deviations are only prominent in the vicinity of the twin boundary and decay fast within a short distance. In fact, a similar decay has also been reported for a simulated twist grain boundary in LLC within the Ginzburg–Landau scheme (Belushkin & Gompper, 2009 ▸).

It is interesting to note that the opposite sides of the partitioning layer can be asymmetric, hence one side can be more strongly perturbed by twinning than the other. One sees in Fig. 2 ▸(*b*) that the intersection of a {111} + 0.5 plane and the silica wall (tracing the partitioning layer) consists of rings arranged on a triangular lattice. The inner and outer circles of a ring correspond to larger and smaller necks in the CMC model, respectively. Clearly, with larger neck radius, the surface is less perpendicular to the twin boundary, hence larger perturbation is entailed. An obvious disparity between the CMC pair with regard to the intersection angles is also shown in Fig. 2 ▸(*d*) at *x* = 0.5.

## 5. Extrapolation of the theory to G-twins   

**Figure 3:**
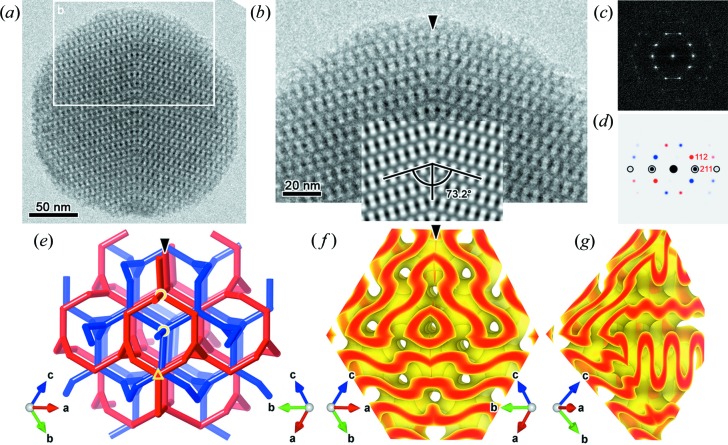
HRTEM image and structural models of a G-twin boundary. (*a*), (*b*) HRTEM images taken along the common 〈
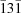
〉 axis, showing a {211} twin boundary. The inset in (*b*) shows a hypothetical image obtained by mirroring a simulated HRTEM image with an offset value of 0.5. (*c*) The Fourier transform of the HRTEM image in (*b*). (*d*) A superposition of two simulated ED patterns (red and blue) in which the 211 reflections of both domains overlap with each other (black). (*e*) Skeletal graphs for the G-twin boundary in which the 3- and 5-connected vertices are highlighted with a yellow triangle and circles, respectively. (*f*) A 3D model of the G-twin boundary obtained by joining two 3D reconstructed copies, shown in (*g*), at {211} + 0.5.

We now extend our discussion to G-BCS. By carefully inspecting HRTEM images of MSC particles identified as G-BCS, a twin boundary, termed G twin, is revealed. HRTEM images [Figs. 3 ▸(*a*) and 3 ▸(*b*)] taken from a common 〈311〉 axis show that the twin boundary can be indexed as {211} and that its contact angle with a {110} plane on each side is around 73.2°. In the inset of Fig. 3 ▸(*b*) (and Fig. S7), a simulated HRTEM image of G-BCS, modelled using a 4-term nodal approximation (Ohsuna *et al.*, 2011 ▸), is merged with its mirror image at {211} + 0.5, fairly reproducing the observation. A superposition of simulated ED patterns [Fig. 3 ▸(*d*)] also agrees with the Fourier transform shown in Fig. 3 ▸(*c*). Remarkably, we find that a twin boundary that was reported in a copolymeric G-BCS (Vignolini *et al.*, 2012 ▸) has the same twin orientational relationship as the present one.

**Figure 4:**
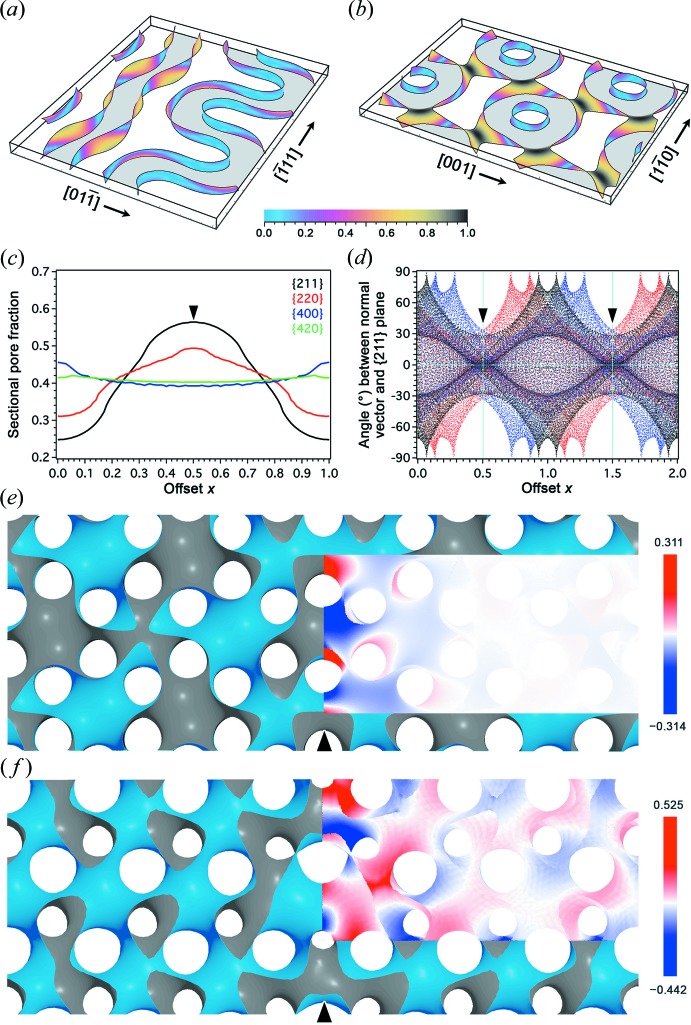
Structural assessment of a G-twin boundary. (*a*), (*b*) Rendering of |**n**_s_·**n**_b_| on the side surfaces of the reconstructed silica wall at {211} + 0.5 and {220} + 0.5, respectively, shown within slab regions of thickness 0.2 in units of the relevant *d* spacings. (*c*) Sectional pore fractions calculated with the reconstructed silica wall, in which the {211} + 0.5 shows the highest contrast. (*d*) Scatter plot of elevation angles of the surface normal vectors with respect to {211} planes plotted against the offset values, for the G surface (black) and its CMC companions (red and blue). (*e*), (*f*) Twin-boundary sections of polysynthetic G twins constructed as minimal and CMC surfaces, respectively, viewed along a common 〈111〉 direction. Colour maps are overlaid to show the minimal distances in units of the *d* spacing from the un-twinned G surface as in Fig. 2 ▸. Note that the surfaces are smoothened at the twin boundaries, while the modification is stronger than in the case of a D-twin boundary.

The two disjoint labyrinths of G-BCS can be described using 3-connected nets whose nodes correspond to the 16*b* positions [for 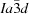] with the site symmetry 32. Both the red and blue skeletons are reflected with respect to the G-twin boundary, yielding 3- and 5-connected junctions on the boundary after fusing every close pair of nodes [Fig. 3 ▸(*e*)]. The modified topology also bears a chirality fault at which the two networks swap their handedness. As in the case of the D-BCS, we adopt the G-BCS that was reconstructed by Han *et al.* (2011*a* ▸) [Fig. 3 ▸(*g*), Table S2 and Fig. S8]. Recall that the side surfaces of the silica wall were approximated as the equipotential surfaces that minimize the Helfrich energy density with κ_2_ = 0, wherein the threshold value was determined to be 53% within the min–max range of the equipotential. We find that two mirrored domains at {211} + 0.5 meet almost smoothly [Fig. 3 ▸(*f*)]. Indeed, the {211} + 0.5 planes of G-BCS are the lowest in terms of the dot product |**n**_s_·**n**_b_| for the reconstructed side surfaces [Fig. 4 ▸(*a*)], accommodating the highest sectional pore fraction [Fig. 4 ▸(*c*)].

If the G surface and its CMC companions are employed as geometrical approximations to the median and side surfaces of G-BCS, respectively, it is again found that elevation angles of the surface normal vectors with respect to {211} are the lowest at half-integer offsets [Fig. 4 ▸(*d*)]. These altogether suggest that {211} + 0.5 is the best candidate of the twin boundary in terms of perpendicularity. Note that the plots in Fig. 4 ▸(*d*) are symmetrical with respect to vertical lines at both integer and half-integer offsets, provided that the blue and red colours are exchanged. This, as in Fig. 2 ▸(*d*), is attributed to the inversion centres (*i.e.* the flat points) lying in the {211} planes at these offsets. In the meantime, the symmetry of each coloured dot distribution at any integer [half-integer] offset position on the horizontal axis is attributed to twofold screw [rotational] axes along 〈110〉 lying in the {211} planes at the relevant offset. Furthermore, an additional mirror symmetry of the plots with respect to the horizontal axis, provided that the red and blue colours are exchanged, is attributed to the *d*-glide planes that orthogonally intersect {211}.

For modelling the G-twin boundary, the modified Willmore functional is reduced to zero using *Surface Evolver* for the minimal (*H*_0_ = 0) and CMC [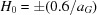] surfaces where *a*_G_ is the cubic lattice constant of the G surface under both the space groups 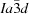 and *I*4_1_32. The model surfaces are bounded by two parallel twin boundaries at a distance comparable to the size scale of the experiment [Figs. 3 ▸(*a*) and 3 ▸(*b*), and Figs. S9 and S10]. Again, the deviations from their un-twinned counterparts are concentrated at the twin boundaries [Figs. 4 ▸(*e*) and 4 ▸(*f*)]. The two CMC surfaces can be swapped by a glide operation that does not change the twin boundaries [as reflected in Fig. 4 ▸(*d*)], hence the two side surfaces of the partitioning layer are perturbed equally.

## 6. BCS twinning as stacking fault   

Defects are no stranger to MSCs. In particular, cage-type MSCs are built of spherical micelle/silica composites in analogy with atomic crystals of atoms. It is then no surprise that twinning has been extensively observed in a number of cage-type MSCs (Miyasaka *et al.*, 2006 ▸; Sakamoto *et al.*, 2009 ▸). In contrast, a collective self-organization of a molecular assembly is essential in the formation of a bicontinuous MSC, where a prior generation of separate building blocks, *i.e.* micelles/silica composites, may not be necessary. In the present synthesis system, the formation of a BCS takes place through a structural relaxation of an inverse bilayer consisting of surfactant molecules during the silica condensation. The surfactant head groups together with additional components, including water and silicate oligomers, form the polar region, which settles down along a TPMS as its median surface. This process should also take place while D and G twins are formed. It has been suggested that bicontinuous phases can be transformed from lamellar phases by forming so-called inter-lamellar attachments (ILAs) between adjacent bilayers (Squires *et al.*, 2002 ▸; Conn *et al.*, 2006 ▸; Tang *et al.*, 2015 ▸). More recently, direct imaging has also shown that ILAs emerge as open necks between parallel planes (Demurtas *et al.*, 2015 ▸).

**Figure 5:**
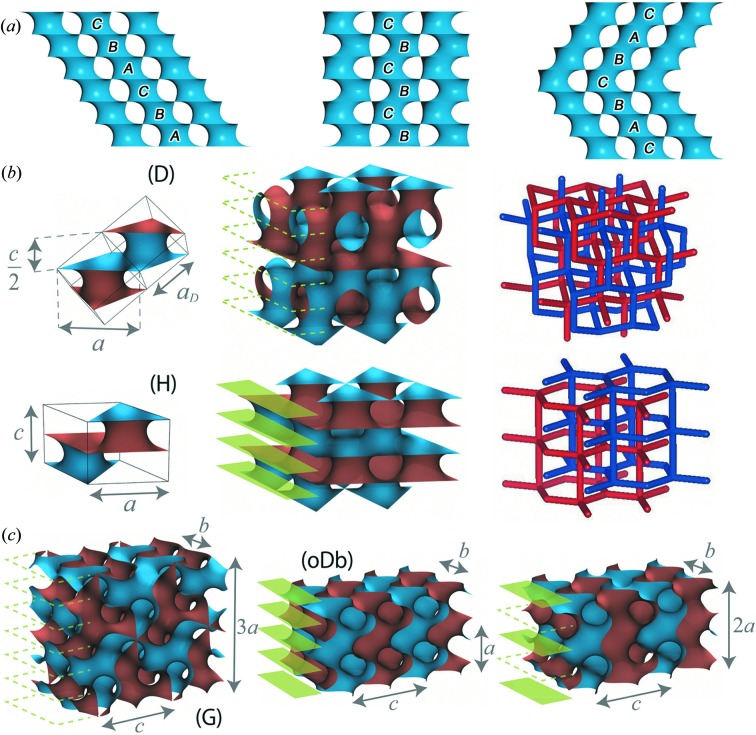
Structural variations associated with D and G twins. (*a*) Possible stacking orders of catenoidal necks. Each catenoidal neck is spanned by two equilateral triangles having a common threefold axis, where the triangles are parallel or anti-parallel if the arrangements of necks in the two adjacent layers are the same (as in the H surfaces) or different (as in the D surface), respectively. For D twins, necks with parallel triangles exist only at the twin boundary. (*b*) The D surface (1st row) and an H surface (2nd row) shown along with their smallest unit cells and labyrinth networks. Reference unit-cell constants are taken from the cubic D surface, such that 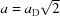 and *c*/*a* = (2/3)^1/2^ where *a*_D_ is the cubic lattice constant. (*c*) The G surface (left) and the first (middle) and second (right) simplest polysynthetic G twins. The reference unit-cell constants are taken from the cubic G surface, such that 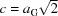, *a*/*c* = (1/3)^1/2^ and *b*/*c* = (3/8)^1/2^ where *a*_G_ is the cubic lattice constant. In (*b*) and (*c*) all the surfaces are oriented with sides being coloured blue and brown. The dashed green lines indicate the candidate positions of twin boundaries (*i.e.* {111} + 0.5 and {211} + 0.5, respectively), while the green plates indicate actual reflection planes. The periods along orthogonal axes are shown with double-sided arrows and their lengths.

The central cavity of our spherical D-twin particles is most likely to be a reminiscence of a sparse centre in a concentric multilayer vesicle in an initial lamellar stage [*cf*. Conn *et al.* (2006 ▸) and Fig. S12]. Then the creation of ILAs initiates the inverse bilayers to rupture so as to diminish the local curvatures, whereby a {111} stacking of necks within each individual D domain is formed. The in-plane positions of necks in each {111} layer of D-BCS define a triangular lattice, while the layer stacking exhibits a cyclic pattern …*ABCABC*… in terms of the neck positions [Fig. 5 ▸(*a*)]. The 3D arrangement of the parallel necks gives a simple cubic lattice (corresponding to the space group 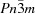). Then, like a twinning in a conventional crystal, a D-twin boundary may arise as a stacking fault, with the ABC pattern reverted at some layer *C* to form a palindromic sequence …*ABCABCBACBA*…. It is also instructive to consider an alternative stacking pattern, …*BCBCBC*…, where the 3D arrangement of parallel necks defines a composite of two simple hexagonal lattices similar to a hexagonal close-packed lattice but with the lattice constant along the threefold axis being halved. The same arrangement is found in Schwarz’s H surfaces (space group, *P*6_3_ *mmc* [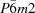]), for which the labyrinths can be represented as interwoven 5-connected nets [Fig. 5 ▸(*b*)]. This in fact allows us to think of an H surface as the simplest polysynthetic D twins, with the shortest distance between adjacent twin boundaries. The neighbourhood of a D-twin boundary hence resembles a slab of an H surface.

Surprisingly, the ILA formation finds correspondence in differential geometry. Traizet constructed virtually all TPMSs near the so-called ‘catenoid limit’ by periodically opening nodes among horizontal planes (Traizet, 2008 ▸). Here, ‘opening nodes’ means a desingularization of singular points (*i.e.* nodes) into small catenoids, in analogy with the opening of ILAs into necks. In particular, Traizet shows the necessity of a balanced condition, namely that all necks are in equilibrium under effective interactions, which are attractive between necks within the same level while repulsive across adjacent levels, with no interaction across levels farther apart. Their strengths are inversely proportional to the distance between the necks while proportional to their sizes, similar to electrostatic forces in the 2D universe. The balanced condition is satisfied for the D twin at the catenoid limit (Chen, 2019 ▸), ensuring the stability of twinned minimal surfaces, though the proof does not work away from that limit.

The formation of a G-twin boundary is also likely to be related to a structural transformation process. In copolymeric systems, it is reported that the transformation from lamellar to G-BCS passes through an intermediate state known as hexagonally perforated layers (HPL) (Foerster *et al.*, 1994 ▸; Khandpur *et al.*, 1995 ▸). The HPL phase can be considered as a hexagonally modulated lamellar phase maintaining an epitaxial relationship with {211} planes of G-BCS. A transient state like HPL is also likely in the pathway from lamellar to G-BCS in LLC systems, considering that a stacking fault in HPL could lead to a chirality fault that is an essential ingredient of the {211} G-twin boundary.

In fact, G-BCS also allows an ILA argument as in the case of D-BCS (Chen, 2019 ▸). More specifically, the G surface is derived if catenoidal necks are introduced between an infinite array of parallel planes, such that necks on the same layer are arranged on a rhombic lattice along a {110} plane of G. One would then consider a transformation from lamellar to G-BCS, during which a stacking fault might arise to generate another type of twin boundary at a {220} + 0.5 plane. Indeed, the {220} + 0.5 planes are the secondary choice in terms of the perpendicularity principle [*cf*. Figs. 4 ▸(*b*) and S10]. So far, however, no {220} epitaxial relation has been reported in lamellar/G phase transformations but in cylindrical/G phase transformations (Honda & Kawakatsu, 2006 ▸; Schulz *et al.*, 1994 ▸).

Again, it is worth mentioning that the simplest polysynthetic G twins with the shortest distance between adjacent {211} twin boundaries correspond to a TPMS represented in Fig. 5 ▸(*c*) (centre). The latter surface is an orthorhombically deformed D surface (oDb surface), with the space group *Cmme* [*Imma*], for which the Weierstrass parameterization is available (Fogden & Hyde, 1992 ▸). The neighbourhood of the G-twin boundary hence resembles a slab of an oDb surface.

## 7. Crystal distortions associated with twinning   

Our MSC samples often exhibit, albeit with varying amounts, crystal distortions that may be associated with twinning. For instance, in Fig. 1 ▸(*a*), the {111} facets surrounding the central cavity are slightly bent inward (see also Fig. S13). If the central cavity were a regular icosahedron, two adjacent triangular facets would subtend a dihedral angle of 138.19°, whereas the ideal dihedral angle that originates from {111} facets of the cubic D-BCS would be 2 × 70.53° = 141.06° with 70.53° being the dihedral angle of a regular tetrahedron. The angle mismatch could be absorbed if the inner facets are bent outward, which opposes our observation.

HRTEM images [Figs. 1 ▸(*b*) and S13] around a D-twin boundary are closely inspected to reveal some crystal distortions that might be responsible for the unusual bending. It is observed near the twin boundary that (i) the period normal to the twin boundary is enlarged by ∼4.5% and (ii) that the periods along the twin boundary shrink by ∼1%. In a sample with thicker shell, the shrinkage along the twin boundary is even larger, leading to more pronounced bending (Fig. S14). On the other hand, the HRTEM image of the G-twin boundary hardly shows bending of the crystal plane, probably because of the small particle size (Fig. S15). Still, the periodicity normal to the twin boundary is enlarged by ∼5.5%.

Generally speaking, the geometry of a BCS is dominated by the geometry of the underlying molecules, which in our case are surfactants. The surfactant packing parameter, 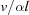, is often used to characterize the molecular shape of surfactants, where *v* is the volume occupied by the hydro­phobic chain, α is the interfacial area per surfactant head group and *l* is the effective length of the hydro­phobic chain. Following the work by Hyde (1989 ▸), it can be argued that the median surface of an inverse bilayer is modelled as a minimal surface for which the area-weighted average of Gaussian curvature is written as 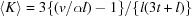, where *t* is the half thickness of the polar region (including ionic head groups, water and silicate oligomers) (Hyde, 1989 ▸). Note that this theory assumes that the curvature fluctuation along the surface is small enough.

**Figure 6:**
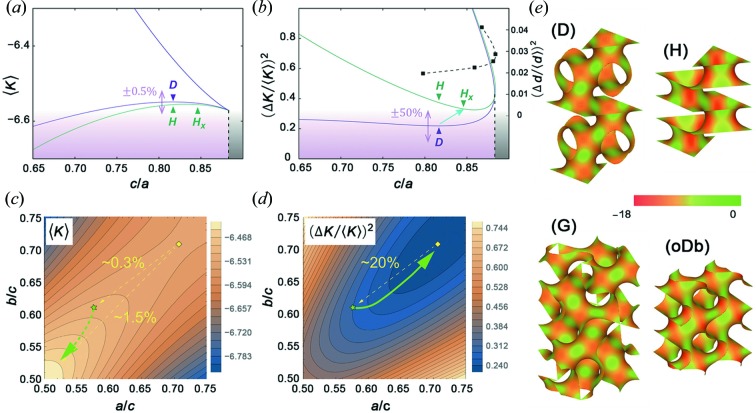
Curvature contrasts at the D- and G-twin boundaries. (*a*), (*b*) Averaged Gaussian curvatures, 〈*K*〉, and the corresponding relative variances, Δ*K* ^2^/〈*K*〉^2^, plotted against *c*/*a* for the rPD and H surface families. The rPD family is obtained by rhombohedrally deforming the D surface. The surfaces in (*a*) are scaled such that the volume per fundamental patch (equivalent to the primitive unit-cell volume, 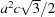) is 2. The two (*i.e.* upper and lower) curves for each family correspond to two distinct minimal surfaces existing for given unit-cell dimensions. As *c*/*a* is decreased, the upper branch approaches the catenoid limit argued by Traizet (Traizet, 2008 ▸). In (*b*), five sampling data of the relative variance of the distance function, *d*, for the H-surface family (Schröder-Turk *et al.*, 2006 ▸) are plotted as black squares with an interpolating dashed curve. (*c*), (*d*) The same quantities as shown in (*a*) and (*b*) but plotted against *a*/*c* and *b*/*c* for the oDb family. The surfaces in (*c*) are scaled such that the volume per fundamental patch (equivalent to the primitive unit-cell volume, *abc*/2) is 2. The position of the reference unit cell [Fig. 5 ▸(*c*), centre] is indicated with a green star, whereas the position of the cubic D surface is indicated with a yellow diamond. For the G surface, 〈*K*〉 is slightly (∼1.2%) above the surface in favour of a deformation along the dotted green arrow, whereas Δ*K* ^2^/〈*K*〉^2^ is beneath the surface by ∼20%, coinciding with that of the D surface (yellow diamond) in favour of a deformation along the solid green arrow. (*e*) Colour maps showing the distributions of the Gaussian curvatures along the D, H, G and oDb surfaces with undistorted unit-cell dimensions, showing enhanced curvature contrasts for the H and oDb surfaces.

Recall that the median surface of twinned D or G surfaces locally resembles an H or oDb surface, respectively, at the twin boundary. All these four minimal surfaces are surfaces of genus three (*i.e.* *g* = 3). It then follows from the Gauss–Bonnet theorem that 〈*K*〉 = 4π(1 − *g*)/*A* = −8π/*A*, where *A* is the surface area per unit cell of the oriented surface. Figs. 6 ▸(*a*) or 6 ▸(*c*) show that 〈*K*〉 is enhanced by ∼0.2% in H compared with D or by ∼1.2% in oDb compared with G, respectively, provided that the reference lattice parameters [*i.e.* *a, b* and *c* in Figs. 5 ▸(*b*) and 5 ▸(*c*)] are fixed. The surface area per unit cell is reduced accordingly. The formal reinforcement of curvature at the twin boundary is, however, so small that it can be readily amended by swelling the unit cell only slightly. Moreover, Figs. 6 ▸(*a*) or 6 ▸(*c*) show that the dependence of 〈*K*〉 on the aspect ratios of the unit cell is extremely weak.

In the meantime, the relative variance of the curvature, Δ*K* ^2^/〈*K*〉^2^ (= 〈*K* ^2^〉/〈*K*〉^2^ − 1), is markedly enhanced in the non-cubic variants as compared with their cubic counterparts, provided that the original aspect ratios of the unit cell (as determined from the reference lattice constants *a*, *b* and *c*) are maintained. Fig. 6 ▸(*b*) shows that Δ*K* ^2^/〈*K*〉^2^ for the H surface doubles that for the D surface. This buildup of curvature fluctuations around the twin boundary, also visualized in Fig. 6 ▸(*e*), would give rise to a strong frustration between the surface and molecular geometries, and is most likely to cost curvature energy. Still, the frustration could be eased by modifying the unit-cell geometry, *i.e.* by increasing *c*/*a* in the direction from H to H_*x*_. Indeed, this is in accordance with the crystal distortion observed experimentally at the D-twin boundary. Similarly, the curvature fluctuations at the G-twin boundary are enhanced by about 20% [Figs. 6 ▸(*d*) and 6 ▸(*e*)], which could be eased if the unit-cell geometry is changed along the green arrow in Fig. 6 ▸(*d*). This is again consistent with the observed small increase of *a* relative to *b* and *c* at the twin boundary. For drawing Figs. 6 ▸(*a*)–6 ▸(*d*), all the three quantities, *A*, 〈*K*〉 and Δ*K* ^2^, have been accurately computed for the TPMSs using the Weierstrass integration formulae (Fogden & Hyde, 1992 ▸).

Apart from the local curvature fluctuations, the fluctuations of labyrinthine diameter would also be enhanced strongly by the non-cubic geometries at the twin boundaries (Schröder-Turk *et al.*, 2006 ▸). The latter effect is considered to be the second source of geometrical frustrations associated with the packing inhomogeneity of the surfactant molecules, which need to be stretched or compressed to fill in the given space. Note that at any point *p* on the surface the distance function *d*(*p*) is defined as the radius of the largest sphere that fits within one labyrinth and touches the surface tangentially at *p*. The labyrinthine diameter at *p* is then measured by doubling *d*(*p*). An intriguing fact here is that the increase of Δ*d* ^2^/〈*d*〉^2^ (the relative fluctuation of the labyrinthine diameter) around the D-twin boundary, which resembles an H surface, can be eased not by increasing but by decreasing *c*/*a* [see Fig. 6(*b*)]. This suggests that the packing inhomogeneity is less dominant in determining the surface geometry of the BCS in the present system.

To conclude, we have presented two kinds of twin boundaries in bicontinuous MSCs; namely, one in D-BCS at {111} + 0.5 and the other in G-BCS at {211} + 0.5 planes. The 3D reconstruction of the silica wall has allowed us to elucidate detailed structural characteristics of the twinned BCSs with the aid of geometrical methods to construct and analyze the characteristic surfaces. The finding of primary importance is that the twin boundaries occur at crystallographic planes that intersect the relevant BCS most perpendicularly. Moreover, we presented the novel view that a D-twin boundary is a stacking fault in the arrangement of catenoidal necks. The local geometries of the twinned surfaces have been modelled using non-cubic variants of the TPMSs, whereby the dominant role of the local curvature frustrations in determining the surface geometry has been revealed. The present results may provide further insights into the formation processes of BCSs in natural and synthetic self-assembly systems.

## Supplementary Material

Supporting information. DOI: 10.1107/S2052252519017287/zx5019sup1.pdf

## Appendix A Methods

The MSCs were synthesized in the work by Han *et al.* (2011*a* ▸). The typical synthesis composition is *N*-stearoyl-l-glutamic acid : Brij-56 [C_16_H_33_(OCH_2_CH_2_)_10_OH] : 3-amino­propyl tri­meth­oxy­silane : tetra­ethyl orthosilicate : H_2_O = 1 : *x* : 2 : 15 : 2335, where *x* = 1.45 for D and 1.75 for G. The surfactant-free materials for HRTEM analyses were obtained by calcination at 550°C in air for 6 h.

HRTEM was performed using a JEOL JEM-2100 microscope that was equipped with a LaB_6_ gun operating at 200 kV [spherical aberration coefficient (*C*_s_) of the objective lens = 1.0 mm, point resolution = 2.3 Å]. Images were recorded using a TENGRA CCD camera (resolution of 2304 × 2304 pixels with a 2:1 fibre-optical taper and an effective pixel size of 8 µm^2^) at 50 000–120 000 times magnification under low-dose conditions. HRTEM is the ultimate tool for analysing defects as the structure can be directly observed. Since both phases and amplitudes of crystal structure factors can be obtained by electron crystallography from the Fourier transform of the HRTEM images, the 3D electrostatic potential distribution map can be directly reconstructed to elucidate the characteristic structural features.

To obtain a minimal or CMC surface with *Surface Evolver*, we first prepared an initial surface that is homeomorphic to the target surface by appropriately opening channels between equally spaced parallel planes [following Traizet’s idea; Traizet (2008 ▸)] under prescribed periodic boundary conditions. Then, the surfaces used for the scatter plots of Figs. 2 ▸(*d*) and 4 ▸(*d*) were obtained by minimizing the area under fixed volume ratios between the two labyrinths. For the minimal surface the volume ratio was constrained to be 1, while for each of the two CMC companions it was fixed to be either 3 or 1/3.

We then used translated copies of the unit cell to obtain a thick slab of the minimal surface or either of the CMC surfaces, which we sliced with two parallel planes with selected Miller indices. We imposed free boundary conditions on these planes, so that they would have finally become mirror symmetry planes. The surfaces shown in Figs. 2 ▸(*e*), 2 ▸(*f*), 4 ▸(*e*) and 4 ▸(*f*) were obtained by minimizing the modified Willmore functionals. The prescribed mean curvature *H*_0_ was set to 0 for Figs. 2 ▸(*e*) or 4 ▸(*e*),  for Fig. 2 ▸(*f*) or  for Fig. 4 ▸(*f*), where *a*_D_ or *a*_G_ stands for the cubic lattice constants of the D or G surface, respectively. *Surface Evolver* managed to reduce the modified Willmore functionals down to 10^−25^ even when the twin boundaries were at a distance 15 × *d*_111_ for D twin or 15 × *d*_211_ for G twin, which was comparable to the scale of the experiment.
