# Supplementary material for: Crystal twinning of bicontinuous cubic structures
Source: IUCrJ. 2020 Feb 1;7(Pt 2):228–37. doi: 10.1107/S2052252519017287 (PMC7055389; doi:10.1107/S2052252519017287)
Supplement: Supplementary file 1 [file m-07-00228-sup1.pdf]

IUCrJ

Volume 7 (2020)

Supporting information for article:

Crystal twinning of bicontinuous cubic structures

Lu Han, Nobuhisa Fujita, Hao Chen, Chenyu Jin, Osamu Terasaki and Shunai Che

S1. Classifications of the crystal twinning after Cahn (1954)

S1.1. Growth twins

Growth twins are generated at the solidification front of a liquid-to-solid or vapour-to-solid phase transformation. *Annealing twins* form in polycrystalline metals as one domain grows against another. A *contact twin* arises if one individual grows in contact with another along the twin boundary plane, which often serves as the mirror symmetry plane between the two (*contact reflection twins*). *Repeated* (or *multiple*) *twins* comprise more than two crystal domains that are aligned by the same twin law. If contact twins occur alternately with parallel boundaries they are called *Lamellar twins*, or else *polysynthetic twins* if there are a large number of lamellar domains. If multiple twins are not aligned parallel but cyclically, they are called *cyclic twins*. *Penetration twins* have interfaces which include different lattice planes or which are even irregular.

S1.2. Thermal and Transformation twins

Thermal and transformation twins are generated through solid-to-solid phase transformations. *Thermal twins* occur upon heating a crystal, starting as a stable phase at low temperatures, when mechanical stresses increase as a result of thermal expansion and structural change. Conversely, *transformation twins* form upon cooling a crystal, starting as a high temperature phase, through cooperative atomic movements associated with the phase transformation to the low symmetry phase.

S1.3. Mechanical twins

Mechanical twins are formed through large cooperative atomic movements associated with a martensitic transformation, which is often enforced by external stress. The resulting crystal structure is identical to the original but takes different orientation, while this side of the crystal undergoes a macroscopic change of shape which can be described as a simple shear.

S2. Electron crystallographic study of the mesoporous crystals

HRTEM is the ultimate tool in analysing defects in MCs since it allows a direct observation of the structure. Another advantage over the conventional X-ray crystallography, which is based on diffraction, is that both phases and amplitudes of crystal structure factors (CSFs) can be immediately obtained through the Fourier transform of the HRTEM images of a single domain, thereby the 3D electrostatic potential distribution map can be reconstructed to elucidate the characteristic features of the crystal structure. As electron crystallography has proven to be a powerful method for the determination of mesoporous structures, a 3D reconstruction of the structure was conducted on the basis

of the analysis of the Fourier diffractograms taken from the TEM images along the high-symmetric zone axes.

Table S1 and S2 show the crystal structure factors of G and D. The structure factors are obtained in the HRTEM images in our previous publication from the hollow spheres with multiply twinned D structure and the G structure contains the G-twin (Han *et al.*, 2011a; 2011b). The phase and amplitude of reflections from different projections were extracted and merged into a 3D data set by adjusting the common origin and a normalization process by scaling the amplitudes with common reflections. The electrostatic potential map was obtained through the Fourier synthesis of the crystal structure factors. The interface structure was determined as an equi-potential surface which minimizes the Helfrich energy density on varying potential levels. The determined threshold of D was 75% out of the rescaled 0~100% (min-max value of the electrostatic potential map), and that of G was 53% by minimizing the Helfrich energy density of the boundary (Miyasaka & Terasaki, 2010).

S3. Crystallographic study of the D-twin and G-twin

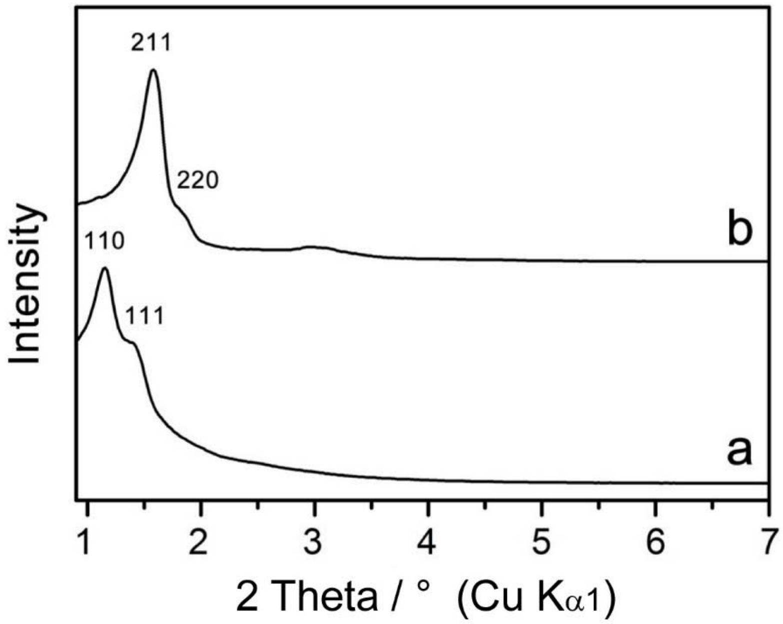

Figure S1 Powder XRD patterns for the mesoporous silica crystals. **a**, D structure with multiply twinned hollow sphere. **b,** G structure contains G-twin. These XRD patterns were recorded with a Phillips PANalytical instrument equipped with monochromator under the conditions Cu $K_{\alpha 1}$ radiation (wavelength 1.5406 Å), 45 kV and 40 mA, in transmission mode at scan rate 0.1° min^{-1} over the range 0.9−7°.

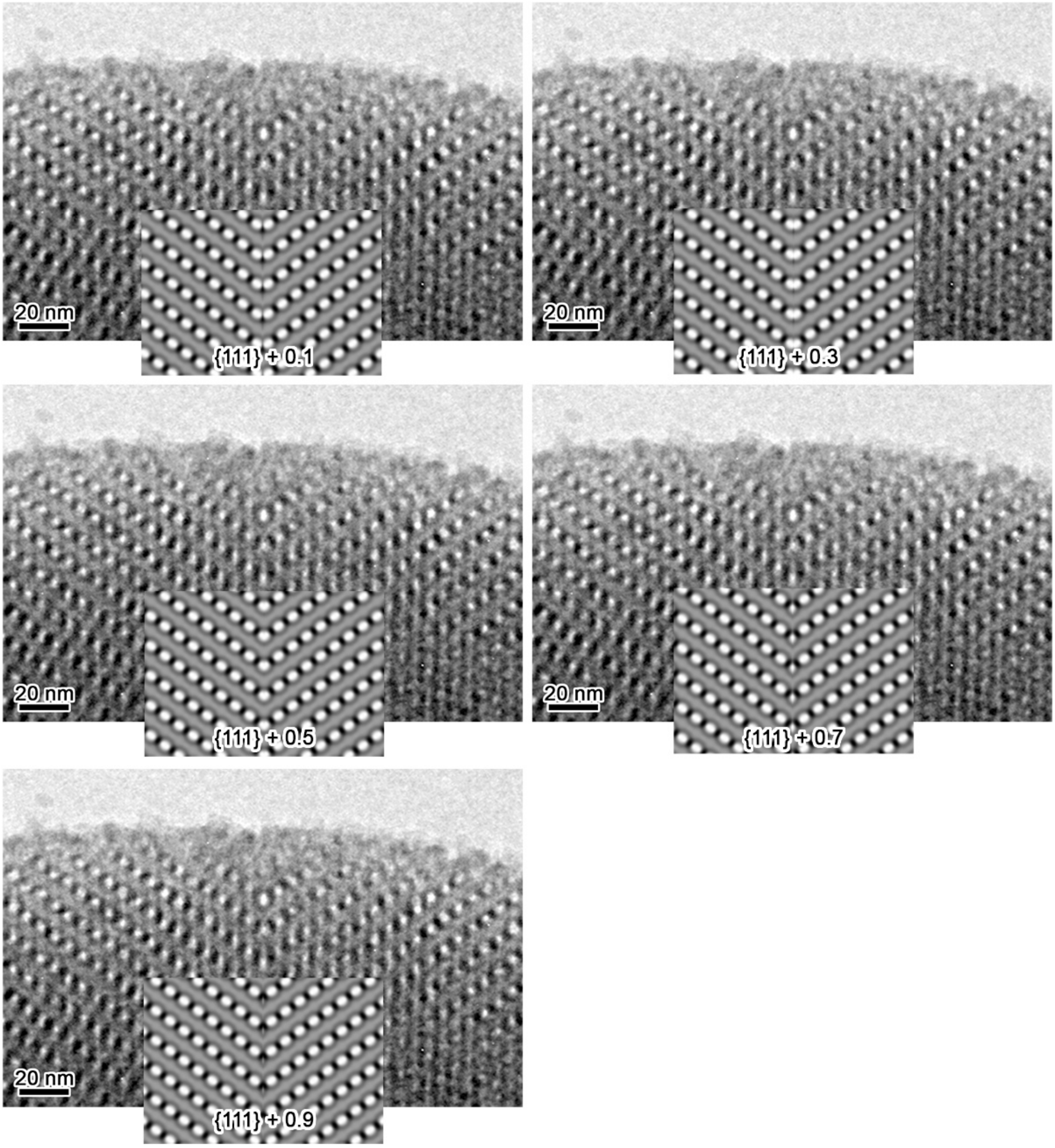

Figure S2 HRTEM image and the simulated image for D twin with different distances from the unit cell origin. Judging from the simulated HRTEM image, the {111}+0.5 plane is most likely the twin boundary.

Table S1 Crystal structure factors (amplitudes and phases) extracted from HRTEM images of D (a = 10.8 nm). In this paper, the reference plane with x = 0 is taken to include flat points on the associated TPMS. For the D surface, for which the space group of the non-oriented [oriented] TPMS is $Pn\bar{3}m$ [$Fd\bar{3}m$], the flat points correspond to the Wyckoff positions 4b [32e] with the site symmetry $\bar{3}m$ [$3m$]. Therefore, phase2 is chosen for the reconstruction.

h	k	l	s*s	d(nm)	Amplitude	Phase1	Phase2
1	1	0	2	7.64	100.00	π	π
1	1	1	3	6.24	54.24	0	π
2	0	0	4	5.40	13.49	0	0
2	1	1	6	4.41	3.40	π	π
2	2	0	8	3.82	0.96	π	π
2	2	1	9	3.60	0.71	0	π
3	1	0	10	3.42	0.30	0	0
2	2	2	12	3.12	0.31	π	π
3	2	1	14	2.89	0.33	0	0
3	3	0	18	2.55	0.20	0	0
3	3	1	19	2.48	0.20	π	0
4	2	0	20	2.42	0.11	π	π
3	3	2	22	2.30	0.12	0	0

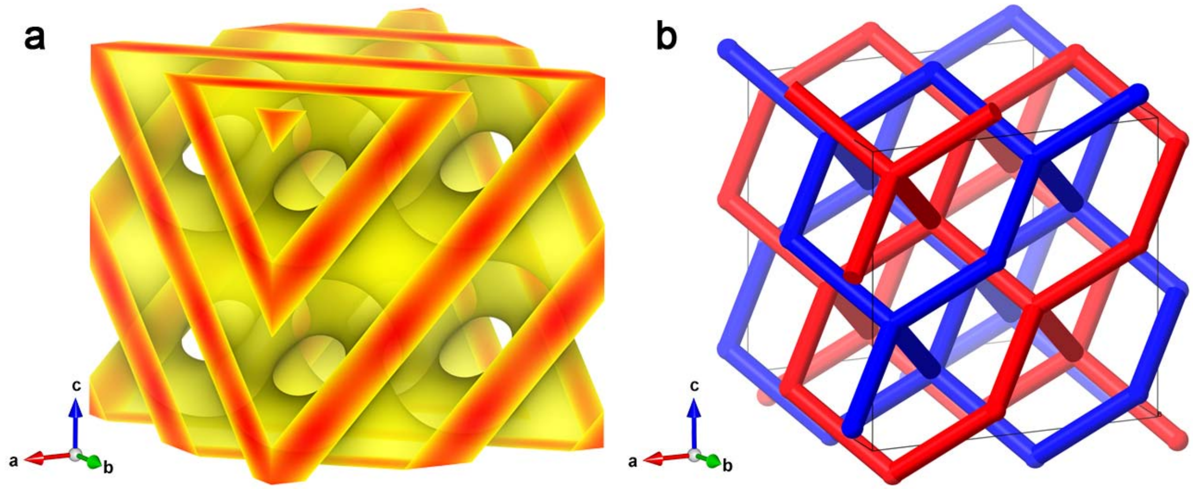

Figure S3 3D reconstruction of the mesoporous silica crystal with D structure. **a**, Reconstructed 3D structure (2×2×2 unit cells). **b**, Skeletal graph (2×2×2 unit cells).

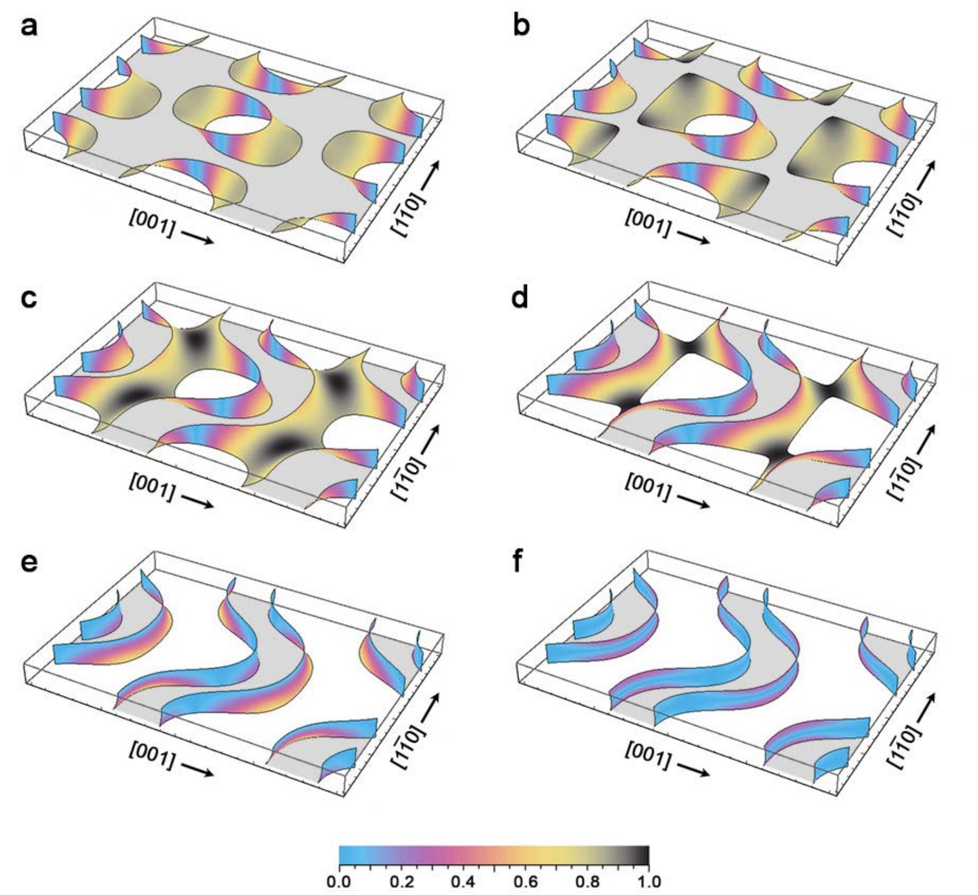

Figure S4 Rendering of |$\mathbf{n}_s \cdot \mathbf{n}_b$| for the D{110} plane. **a**, {110}+0.0, **b**, {110}+0.1, **c**, {110}+0.2, **d**, {110}+0.3, **e**, {110}+0.4 and **f**, {110}+0.5, where a mirror of symmetry is located. The plot region is shown within slab regions of thickness 0.2 in units of the relevant *d*-spacings.

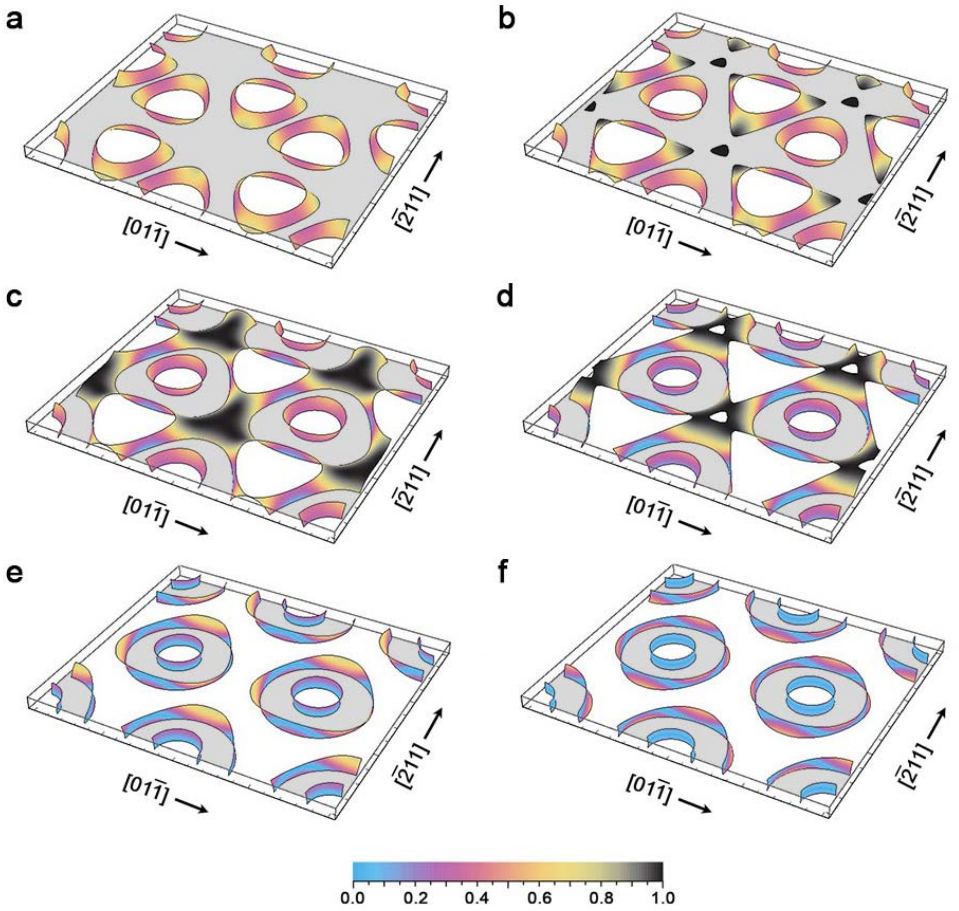

Figure S5 Rendering of $|\mathbf{n}_s \cdot \mathbf{n}_b|$ for the D{111} plane. **a**, {111}+0.0, **b**, {111}+0.1, **c**, {111}+0.2, **d**, {111}+0.3, **e**, {111}+0.4 and **f**, {111}+0.5 (**D** twin) shown within slab regions of thickness 0.2 in units of the relevant *d*-spacings.

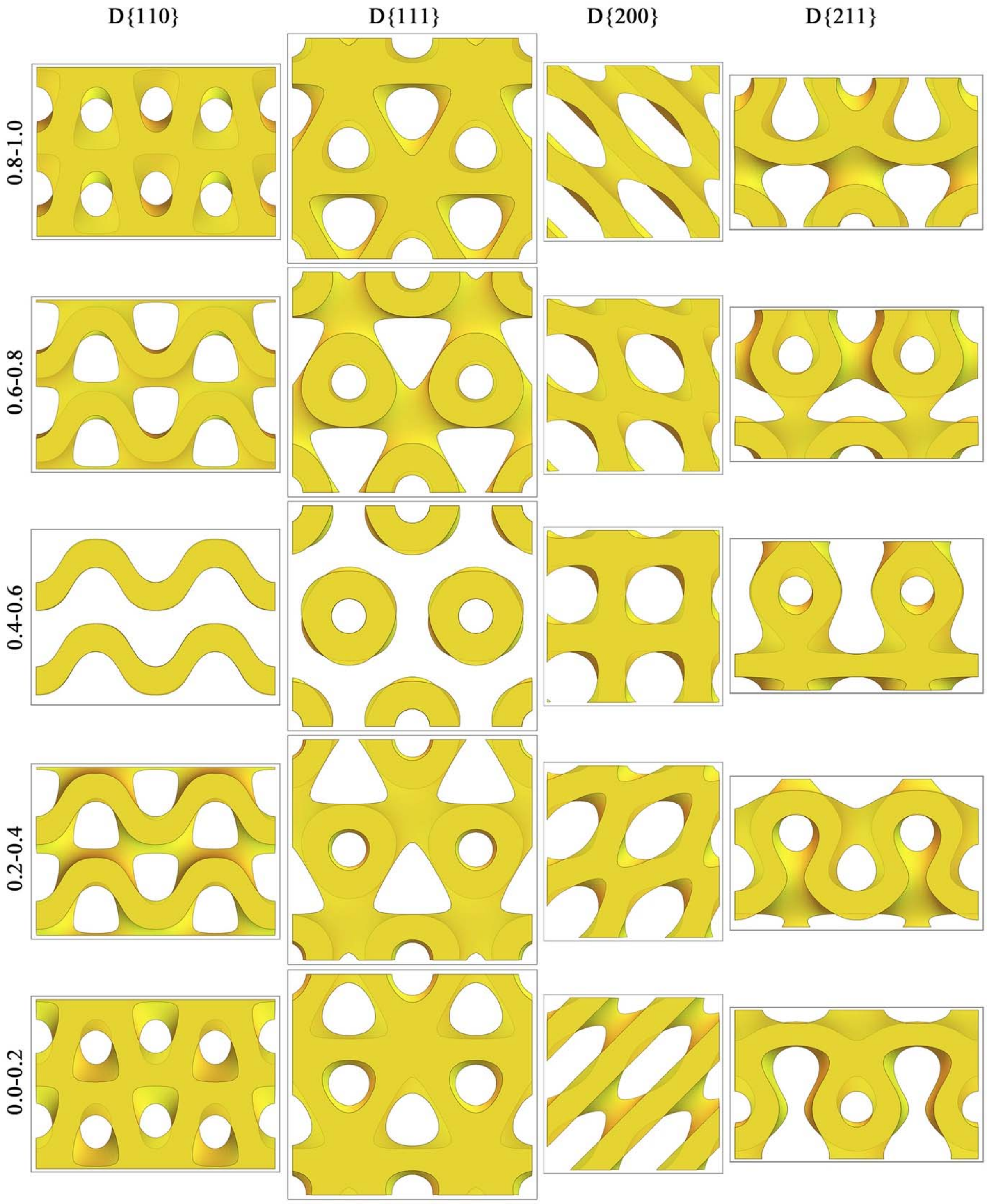

Figure S6 Slice views of D from different orientations. The sectional pore fraction from different orientations can be observed.

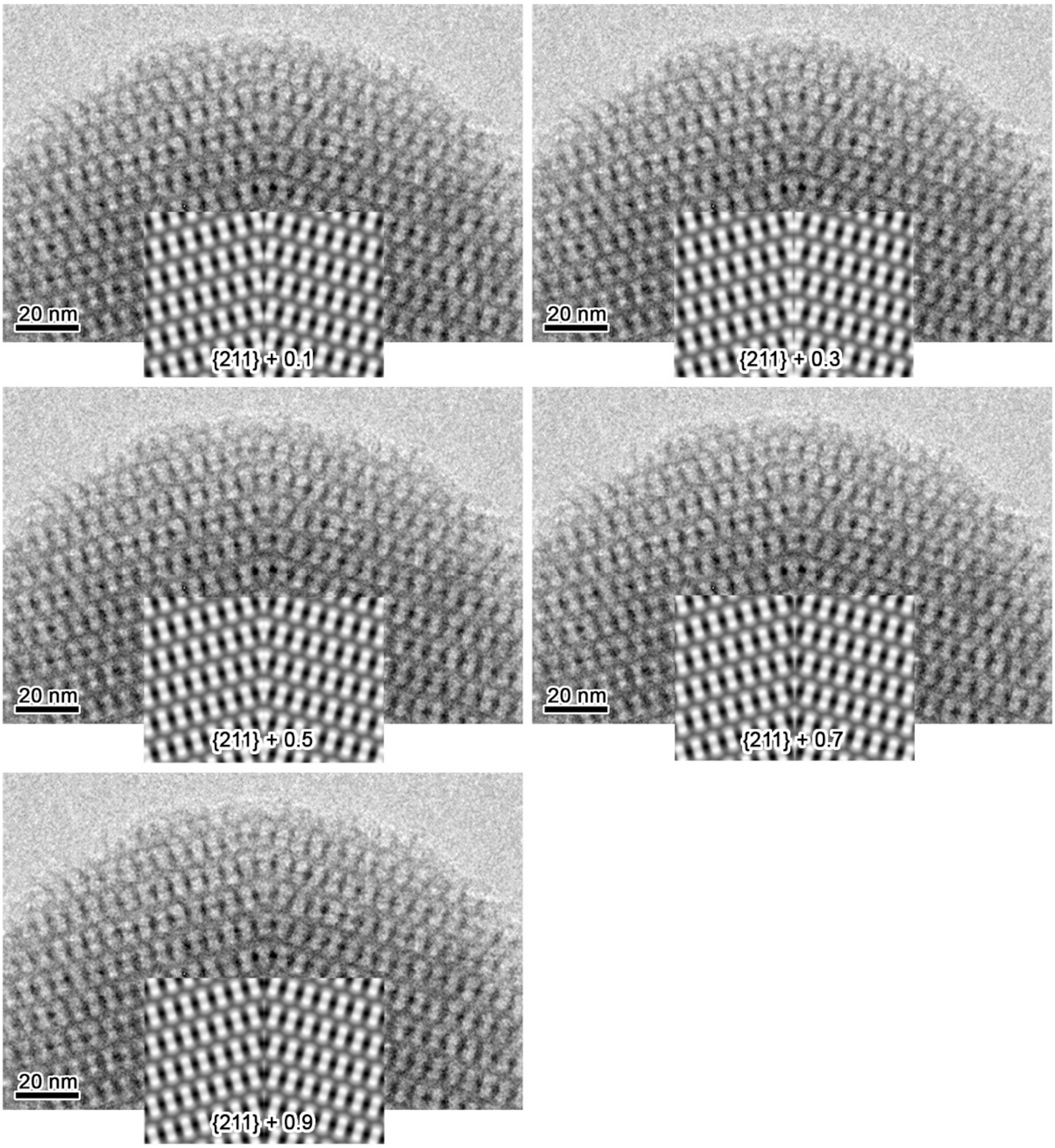

Figure S7 HRTEM image and the simulated image for G twin with different distances from the unit cell origin. Judging from the simulated HRTEM image, the {211}+0.5 plane is most likely the twin boundary.

Table S2 Crystal structure factors (amplitudes and phases) extracted from HRTEM images of G (*a* = 13.6 nm).

h	k	l	s*s	d(nm)	Amplitude	Phase
2	1	1	6	5.55	100.00	π
2	2	0	8	4.81	38.55	π
3	2	1	14	3.64	4.74	0
4	0	0	16	3.40	6.87	0
4	2	0	20	3.04	5.49	0
3	3	2	22	2.90	2.86	0
4	2	2	24	2.78	0.91	0
4	3	1	26	2.67	0.76	π
4	4	0	32	2.40	0.46	0
5	3	2	38	2.21	0.20	0
6	2	0	40	2.14	0.26	π
6	4	0	52	1.89	0.22	π
8	0	0	64	1.70	0.15	0
8	2	0	68	1.65	0.10	0
8	4	0	80	1.52	0.05	0

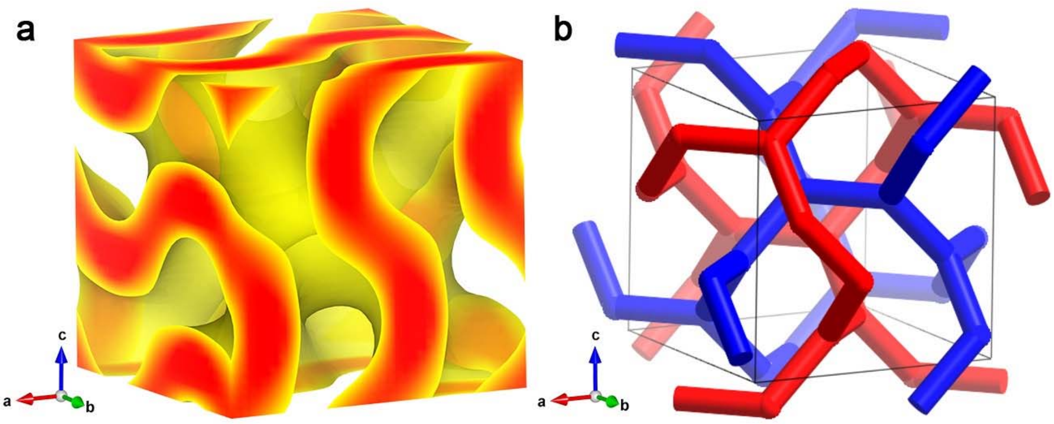

Figure S8 3D reconstruction of the mesoporous crystal with G structure. **a**, Reconstructed 3D structure (1 unit cell). **b**, Skeletal graph of 1 unit cell.

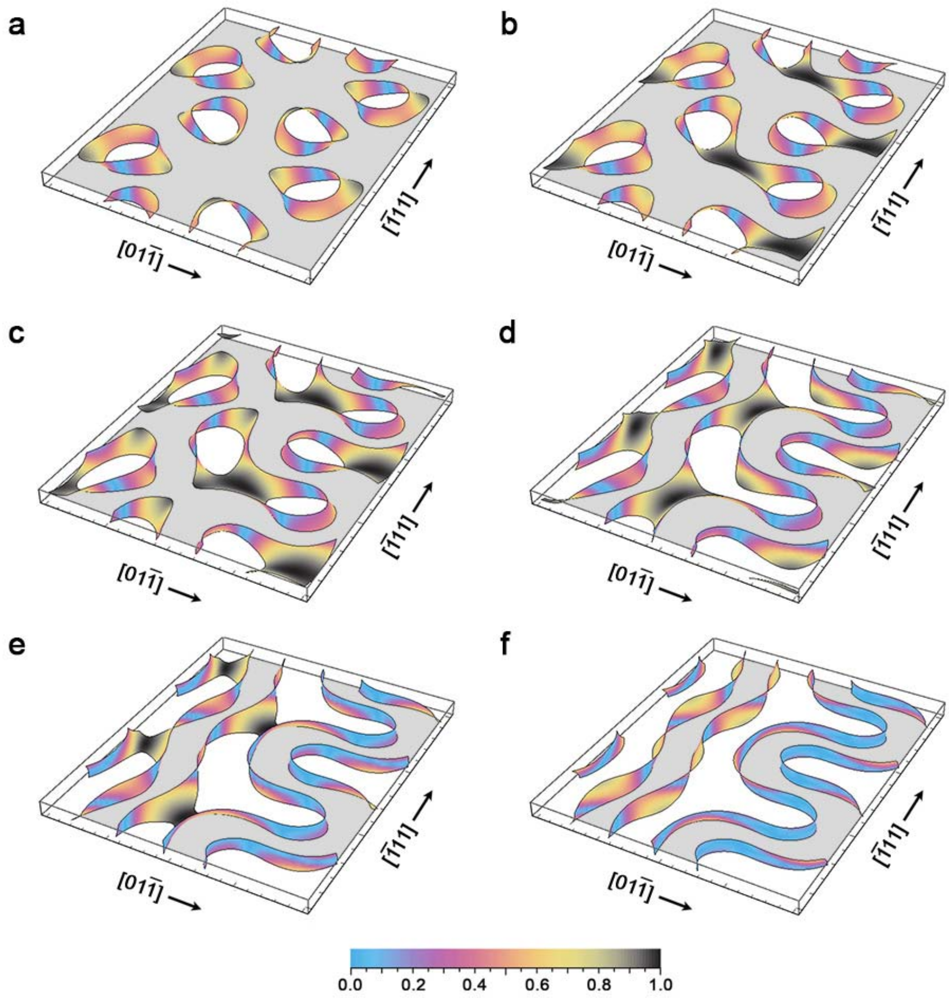

Figure S9 Rendering of $|\mathbf{n}_s \cdot \mathbf{n}_b|$ for the G{211} plane. **a**, {211}+0.0. **b**, {211}+0.1. **c**, {211}+0.2. **d**, {211}+0.3. **e**, {211}+0.4 and **f**, {211}+0.5 shown within slab regions of thickness 0.2 in units of the relevant *d*-spacings.

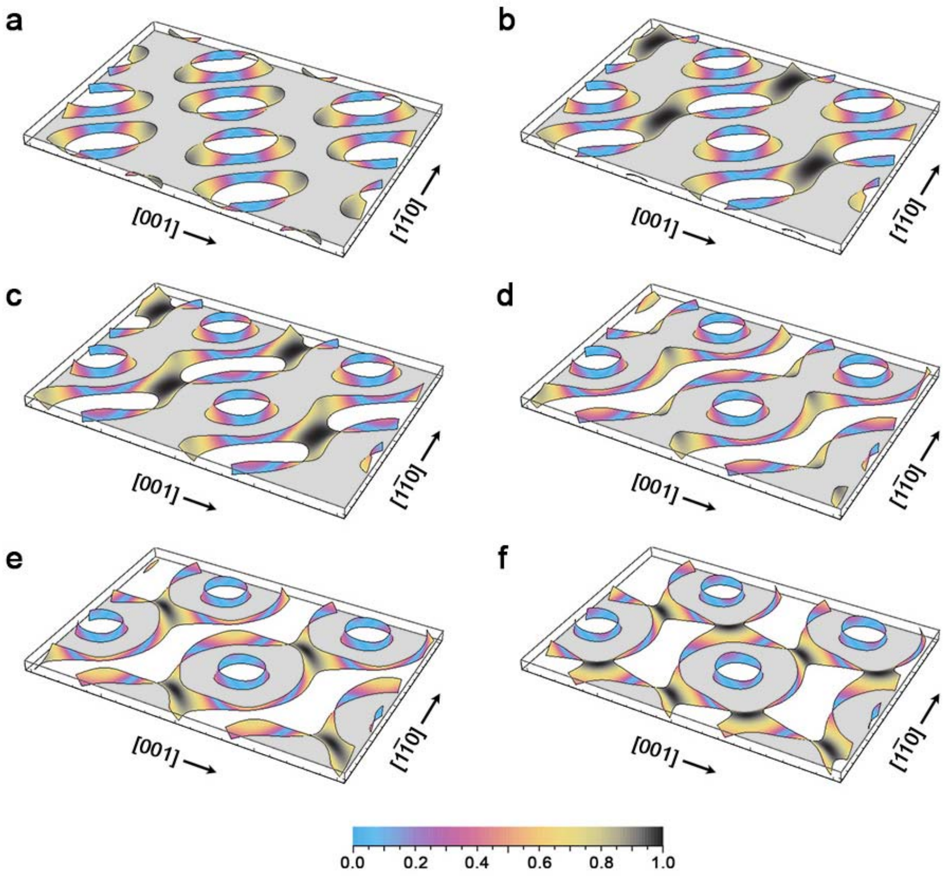

Figure S10 Rendering of |$\mathbf{n}_s \cdot \mathbf{n}_b$| for the G{220} plane. **a**, {220}+0.0. **b**, {220}+0.1. **c**, {220}+0.2. **d**, {220}+0.3. **e**, {220}+0.4 and **f**, {220}+0.5 shown within slab regions of thickness 0.2 in units of the relevant *d*-spacings.

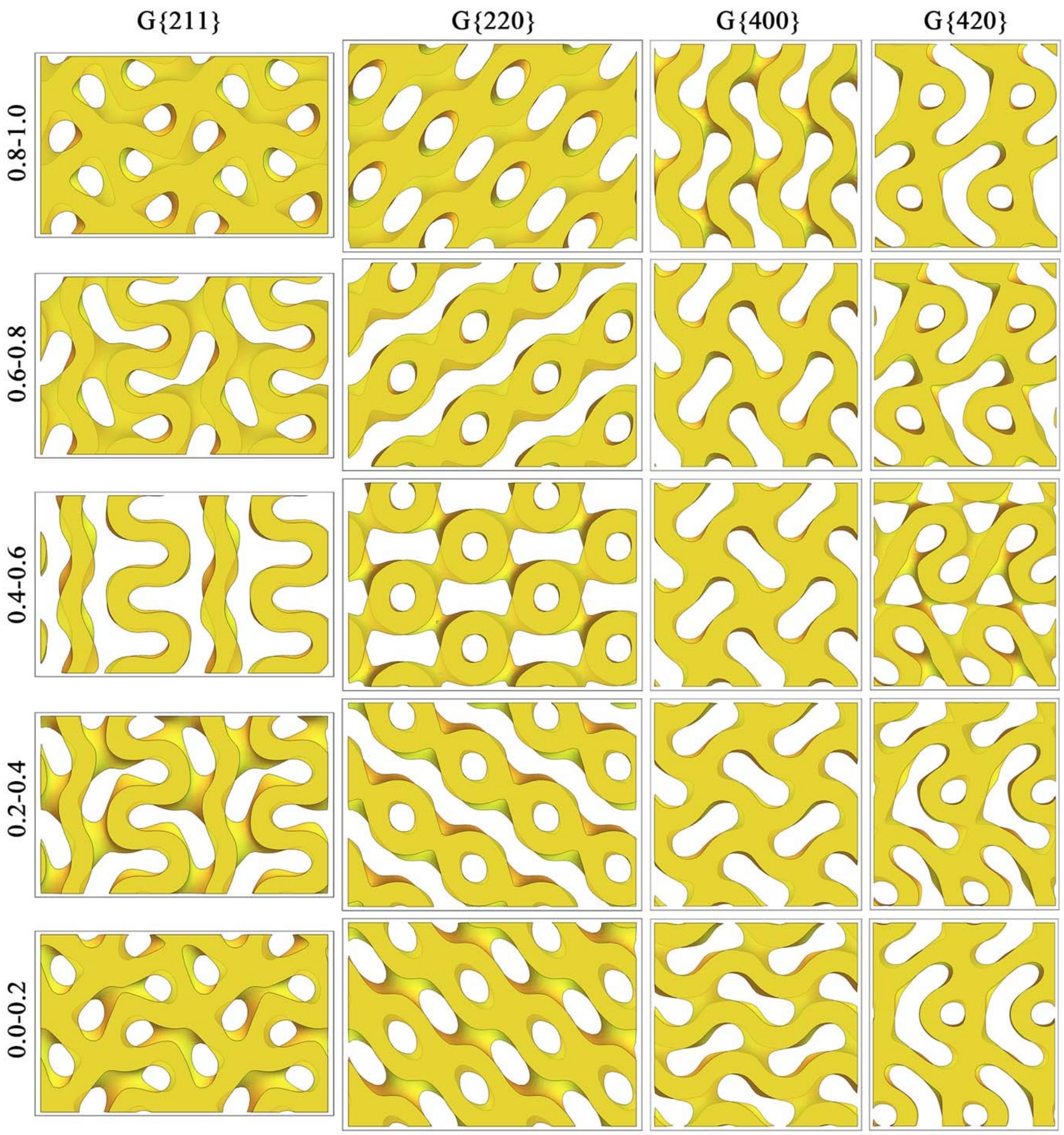

Figure S11 Slice views of G from different orientations. The sectional pore fraction from different orientations can be observed.

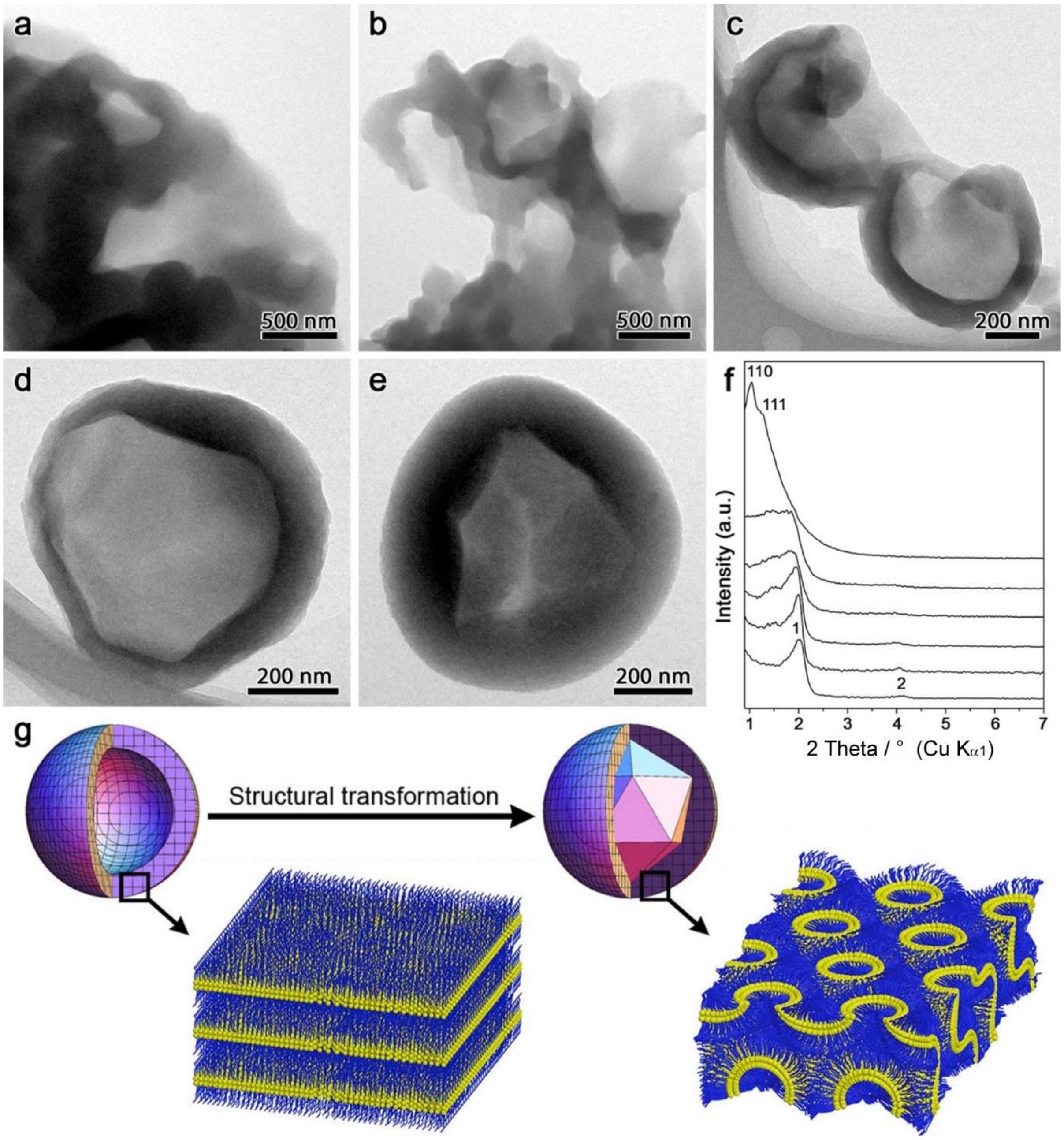

Figure S12 Formation of the D-twin. **a-e**, SEM images of the sample prepared with 1h, 2h, 3h, 4h and 12h, respectively. **f**, Powder XRD patterns of the sample prepared with different reaction time. These XRD patterns were recorded with a Phillips PANalytical instrument equipped with monochromator under the conditions Cu $K_{\alpha 1}$ radiation (wavelength 1.5406 Å), 45 kV and 40 mA, in transmission mode at scan rate 0.1° min^{-1} over the range 0.9−7°. **g**, A schematic drawing of the structural transformation from lamellar to D surface structure.

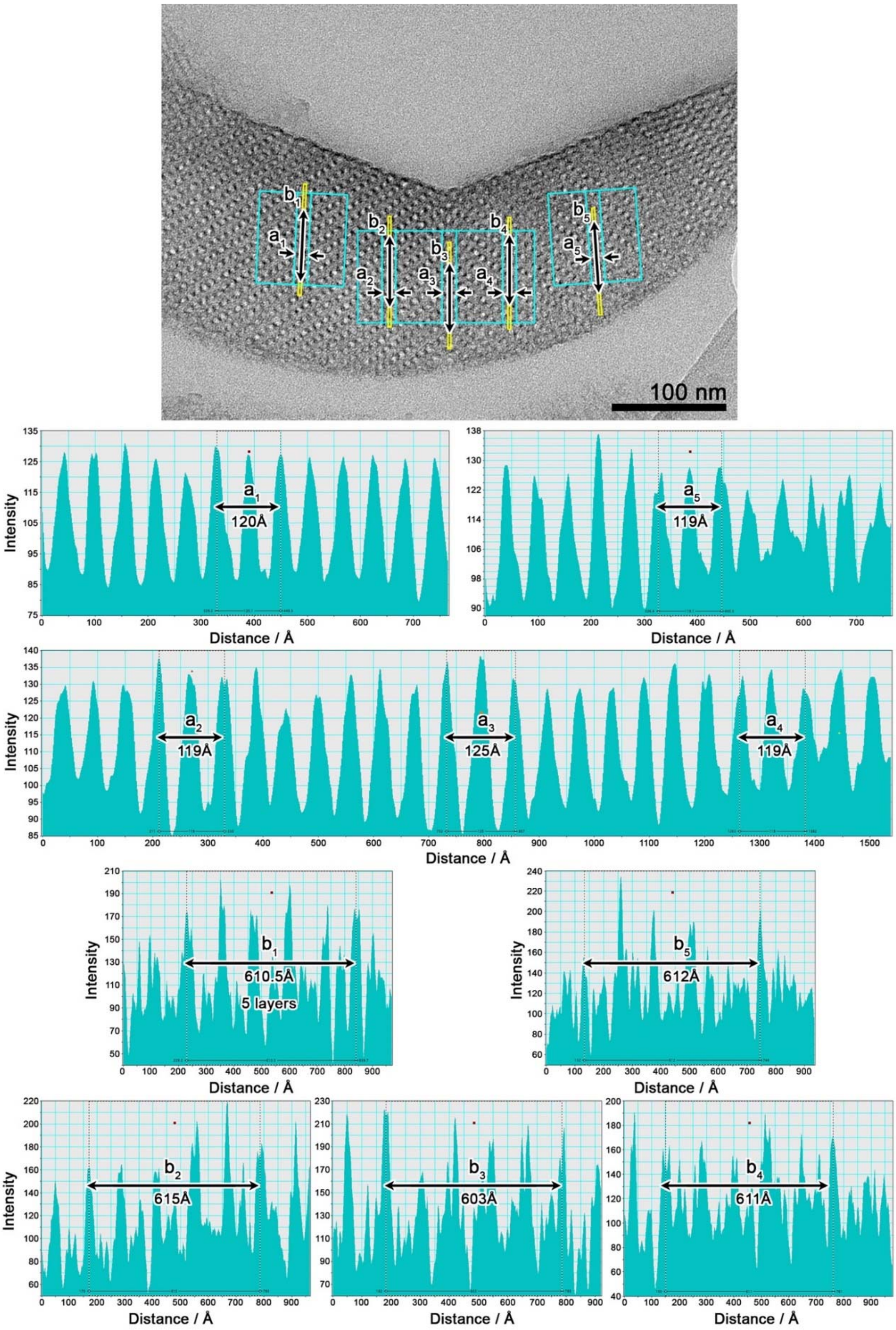

Figure S13 Integrated intensity profile from the HRTEM image of the sliced D-twin.

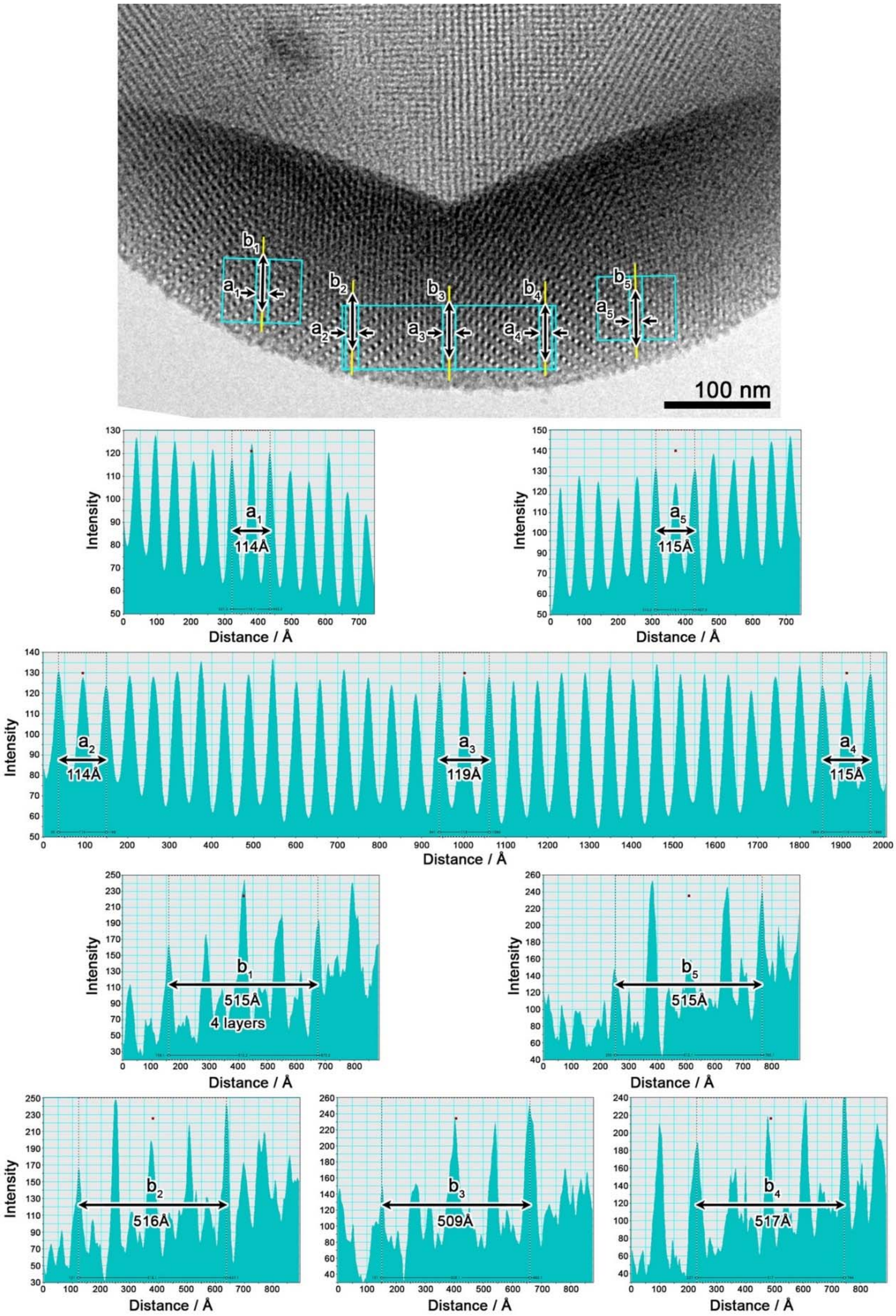

Figure S14 Integrated intensity profile from the HRTEM image of the D-twin.

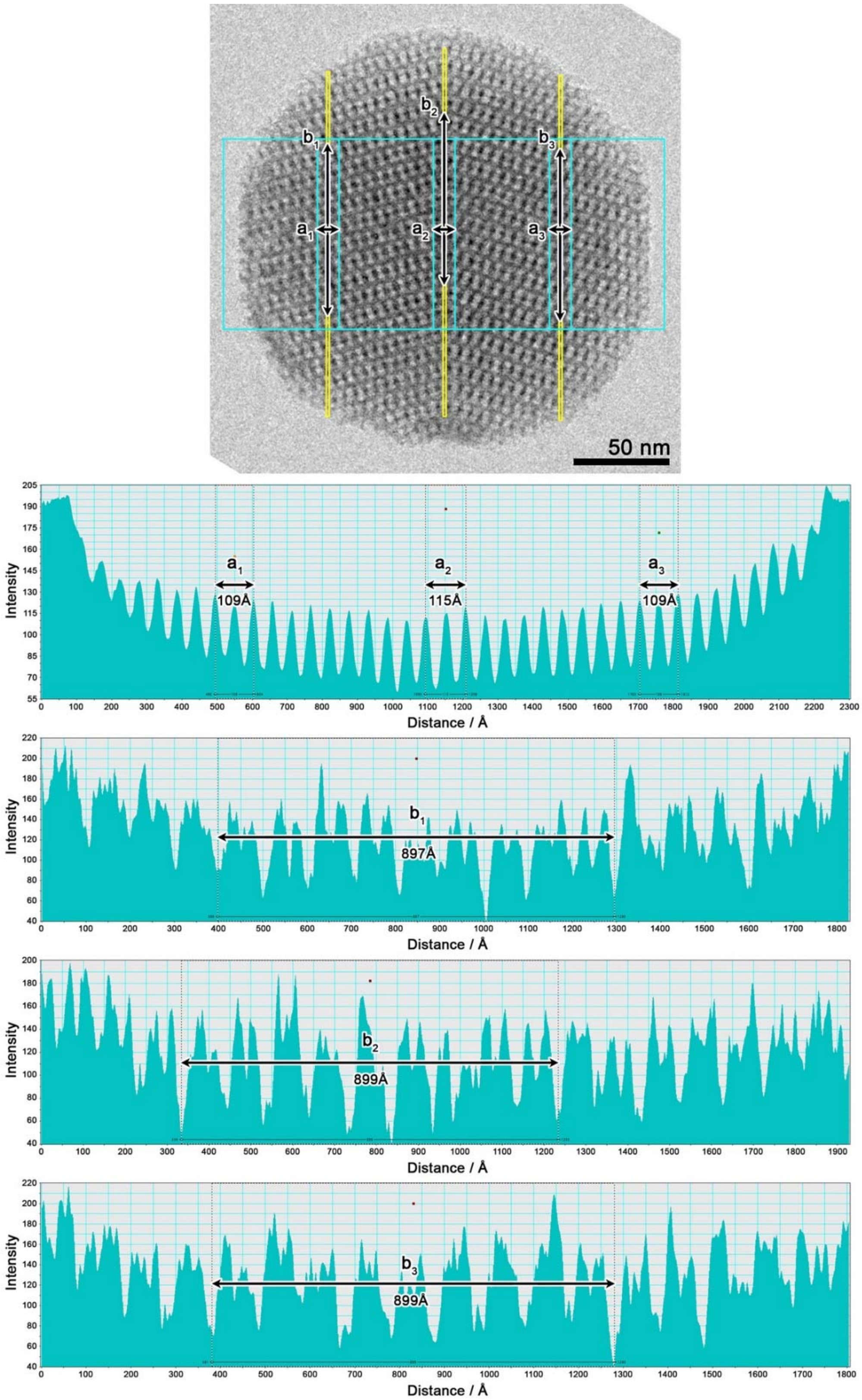

Figure S15 Integrated intensity profile from the HRTEM image of the G-twin.

References

Cahn, R. Twinned crystals. (1954). *Adv. Phys.* **3**, 363–445.

Han, L., Miyasaka, K., Terasaki, O. & Che, S. (2011a). *J. Am. Chem. Soc.* **133**, 11524–11533.

Han, L., Xiong, P., Bai, J. & Che, S. (2011b). *J. Am. Chem. Soc.* **133**, 6106–6109.

Miyasaka, K. & Terasaki, O. (2010). *Angew. Chem. Int. Ed.* **49**, 8867–8871.
